# Supplementary figures and images for: DRfold2 is a deep learning-based tool that enables efficient and accurate RNA structure prediction
Source: PLoS Biol. 2026 Feb 17;24(2):e3003659. doi: 10.1371/journal.pbio.3003659 (PMC12931758; doi:10.1371/journal.pbio.3003659)

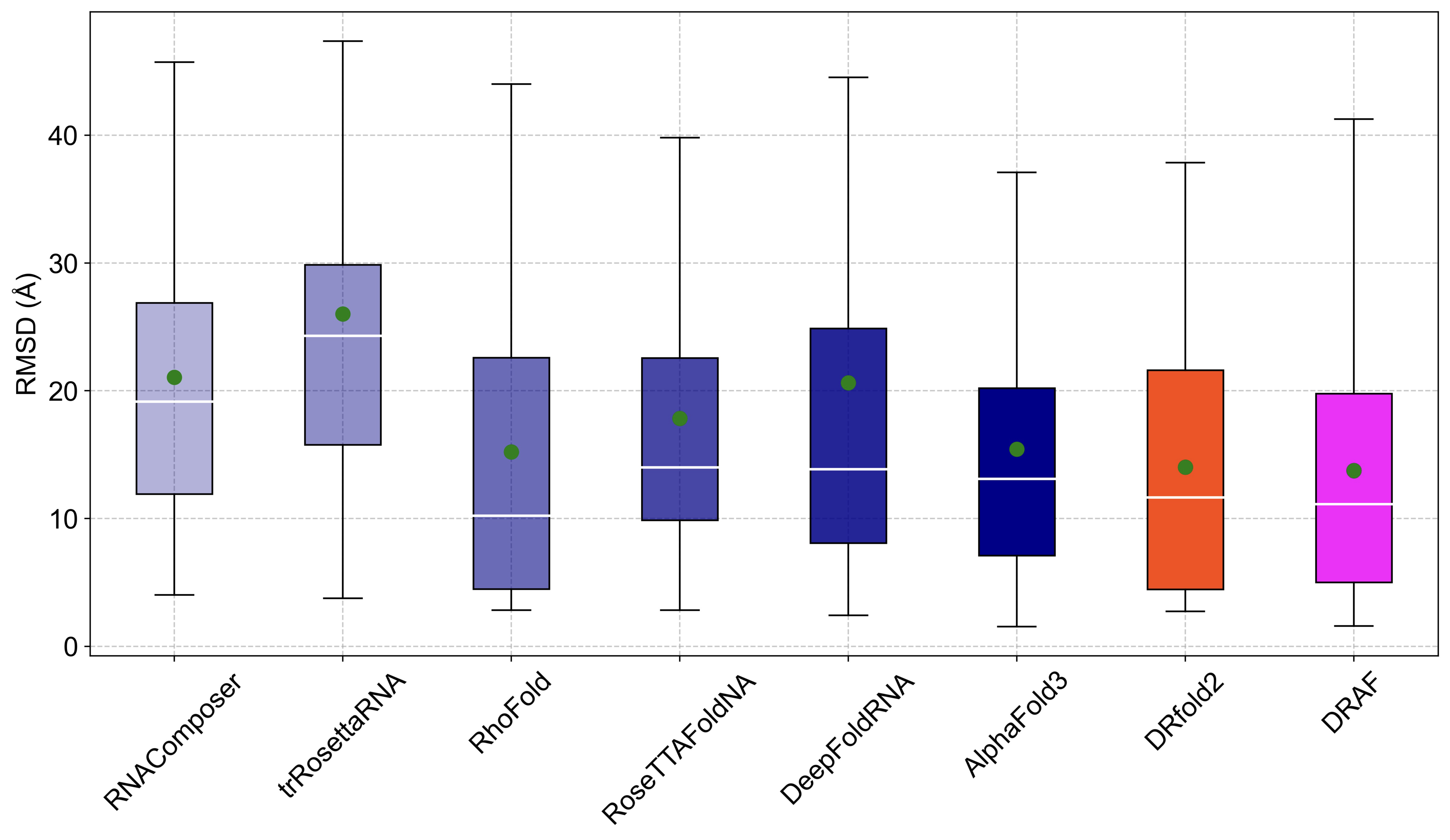

Supplement: S1 Fig — Green points indicate means and white horizontal lines show medians. Underlying numerical data for this figure can be found in S1 Data (see sheets “S1_Data_S1”). (TIF) [file pbio.3003659.s006.tif]

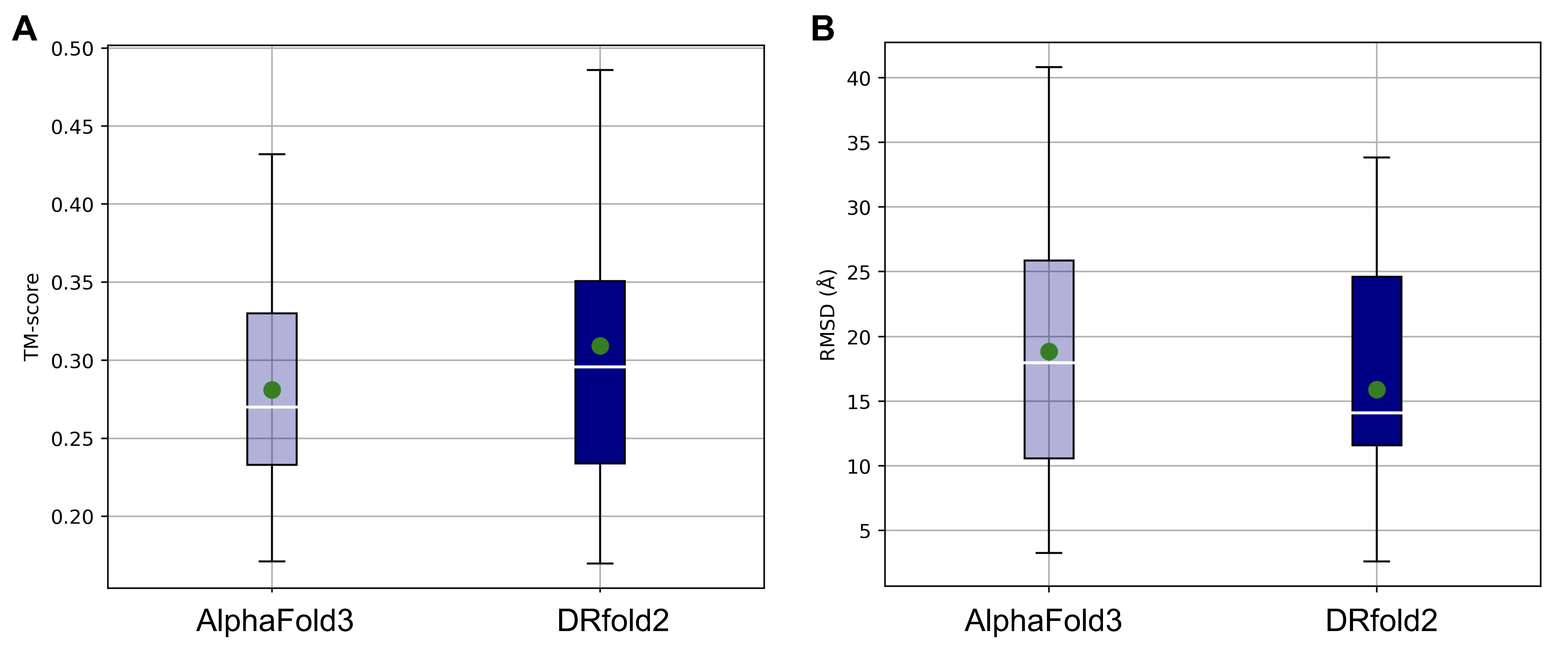

Supplement: S2 Fig — (A) TM-score comparison. (B) RMSD comparison. Underlying numerical data for this figure can be found in S1 Data (see sheets “S1_Data_S2”). (TIF) [file pbio.3003659.s007.tif]

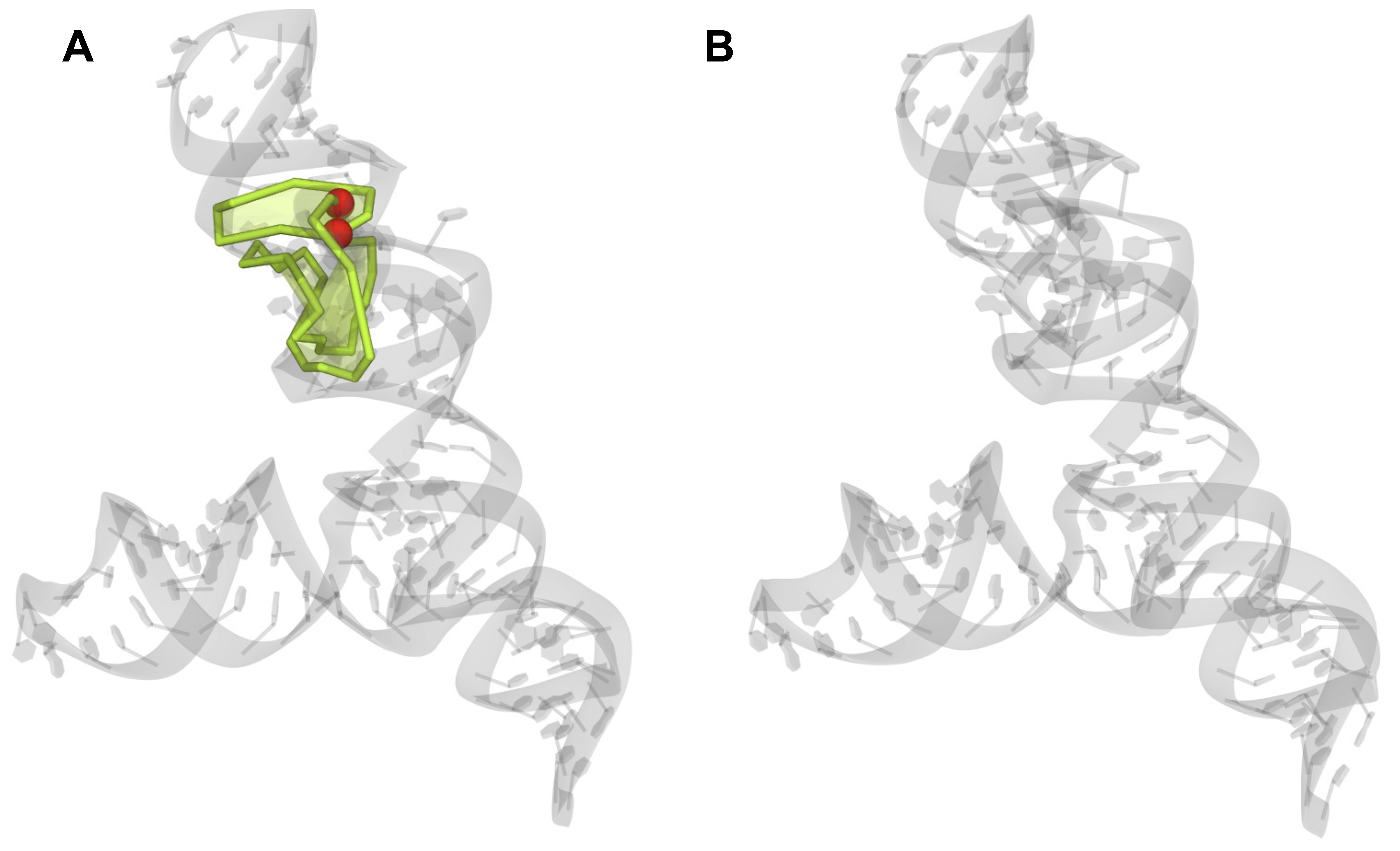

Supplement: S3 Fig — (A) Prediction without refinement. (B) Prediction after refinement. Red dots indicate D&L interlace points. The structural visualizations are screenshots obtained from the RNAspider web server. (TIF) [file pbio.3003659.s008.tif]

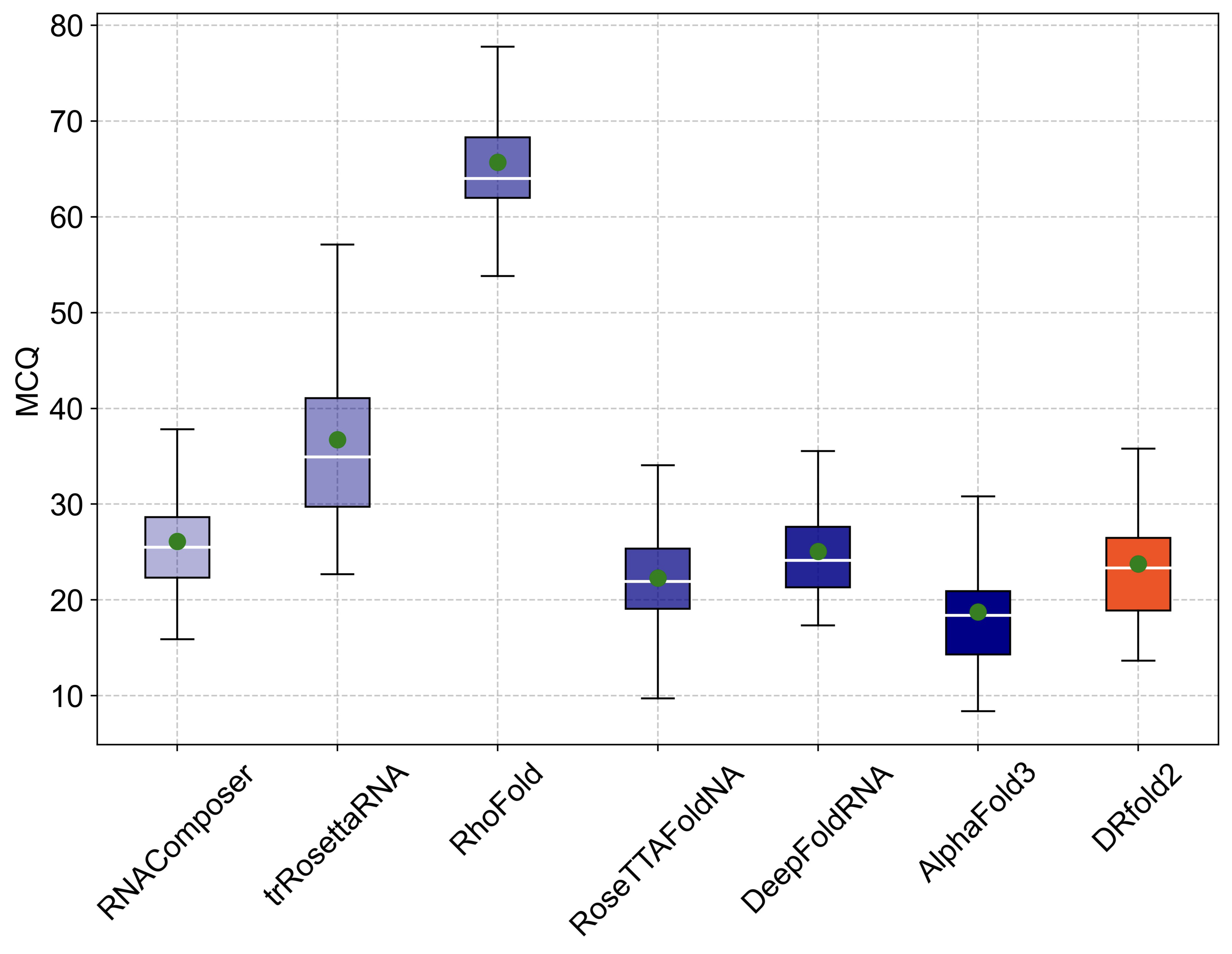

Supplement: S4 Fig — Green points indicate means and white horizontal lines show medians. Underlying numerical data for this figure can be found in S1 Data (see sheets “S1_Data_S4”). (TIF) [file pbio.3003659.s009.tif]

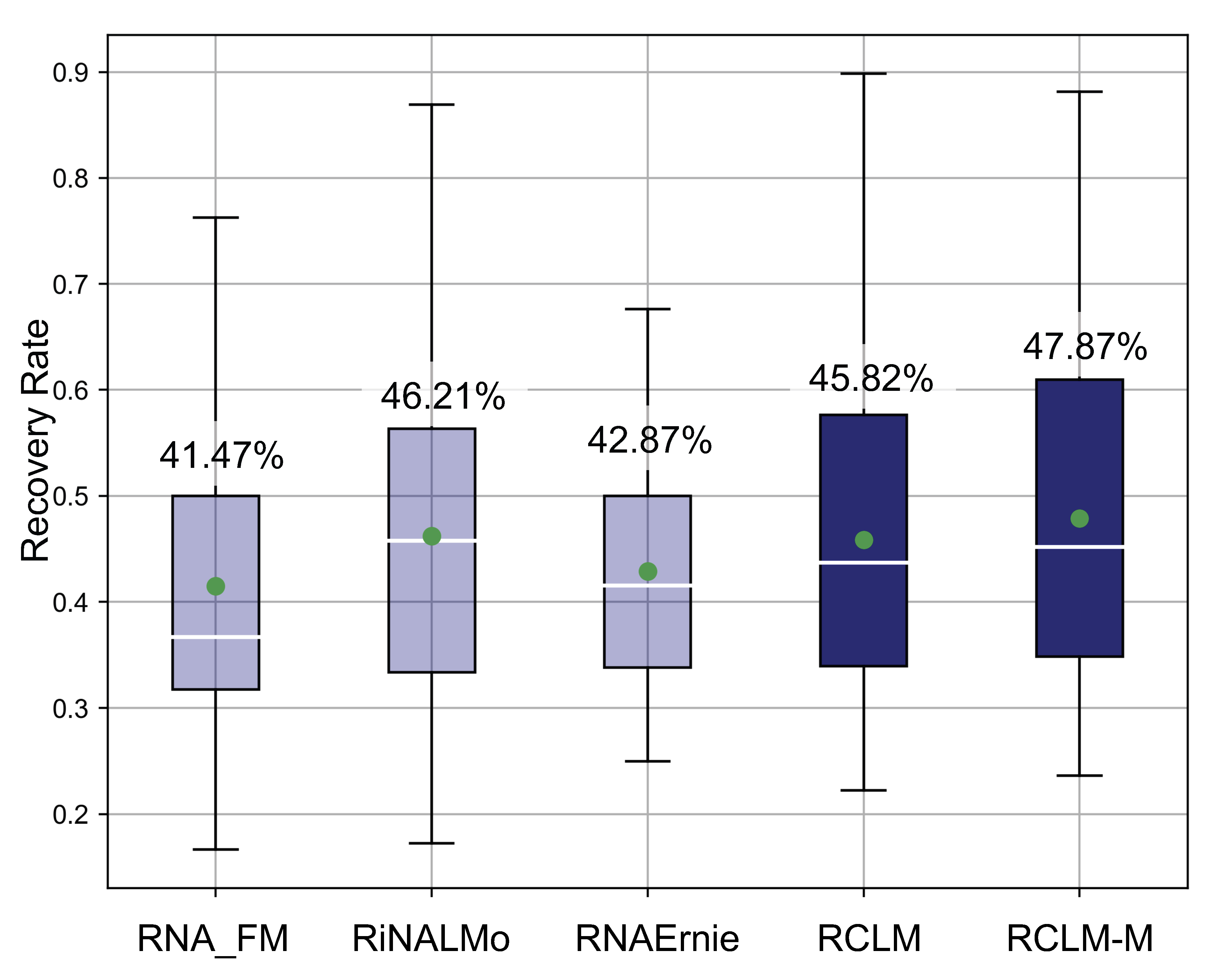

Supplement: S5 Fig — Green points indicate means and white horizontal lines show medians. Underlying numerical data for this figure can be found in S1 Data (see sheets “S1_Data_S5”). (TIF) [file pbio.3003659.s010.tif]

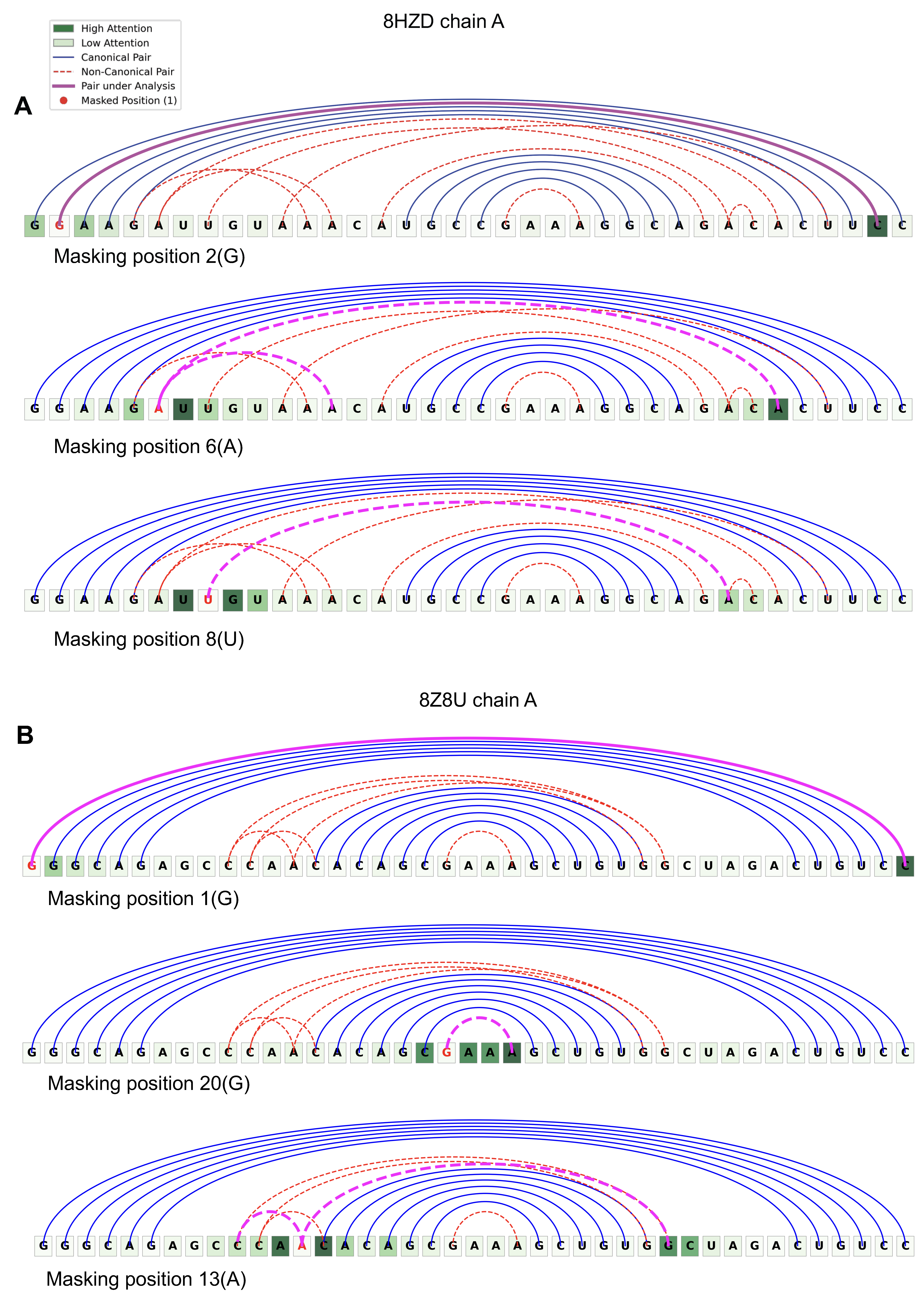

Supplement: S6 Fig — (A) Results for 8HZD chain A. (B) Results for 8Z8U chain A. Nucleotides in red indicate the masked positions. Green nucleotide boxes represent attention magnitudes, where deeper green corresponds to higher attention weights. Blue arcs denote canonical base pairs, red arcs denote non-canonical pairs, and magenta arcs highlight interactions involving the masked nucleotide. (TIF) [file pbio.3003659.s011.tif]

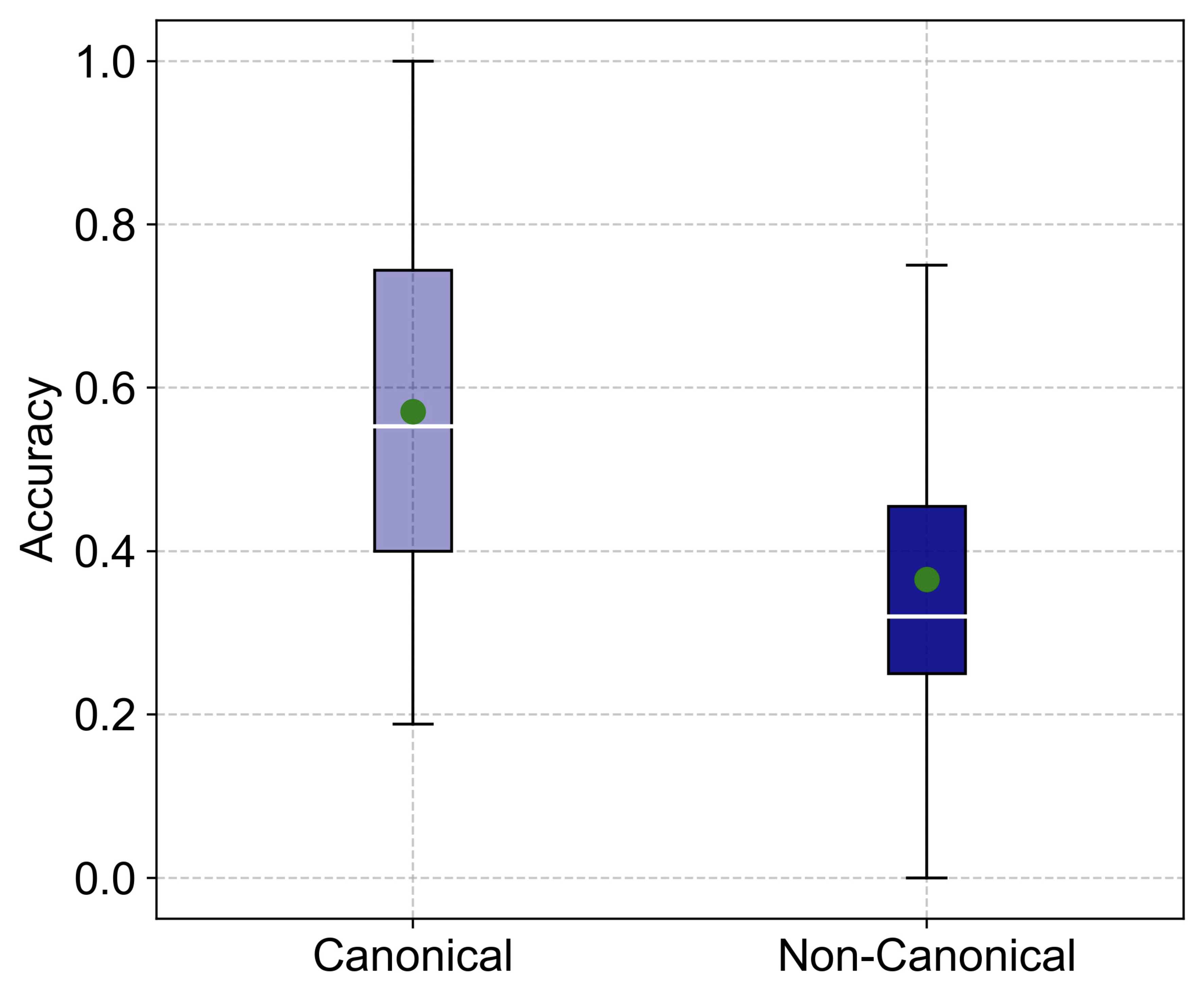

Supplement: S7 Fig — Underlying numerical data for this figure can be found in S1 Data (see sheets “S1_Data_S7”). (TIF) [file pbio.3003659.s012.tif]

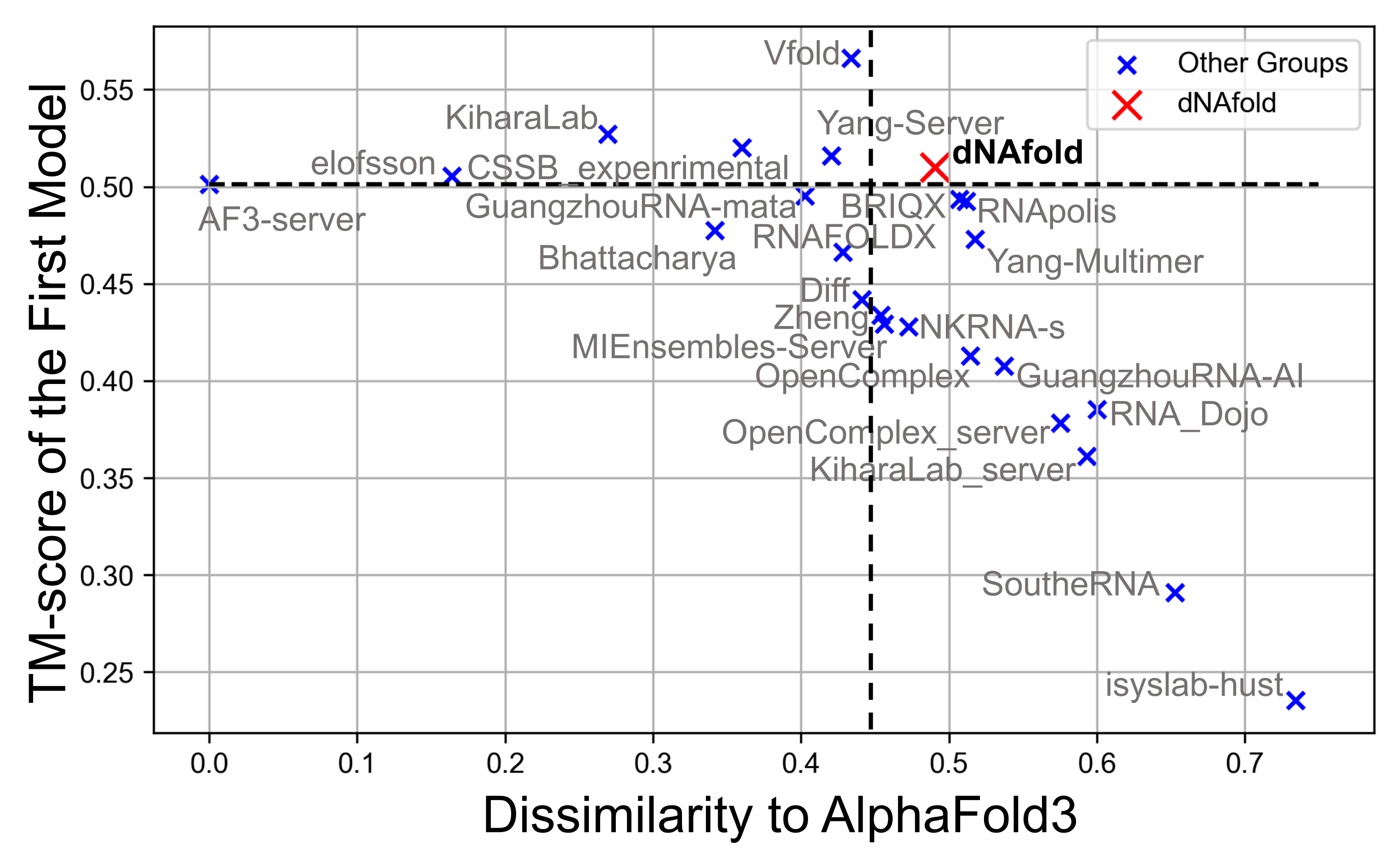

Supplement: S8 Fig — For each group, dissimilarity = 1-TM_M where TM_M is the maximum TM-score between the first model and the five models from AlphaFold3. (TIF) [file pbio.3003659.s013.tif]

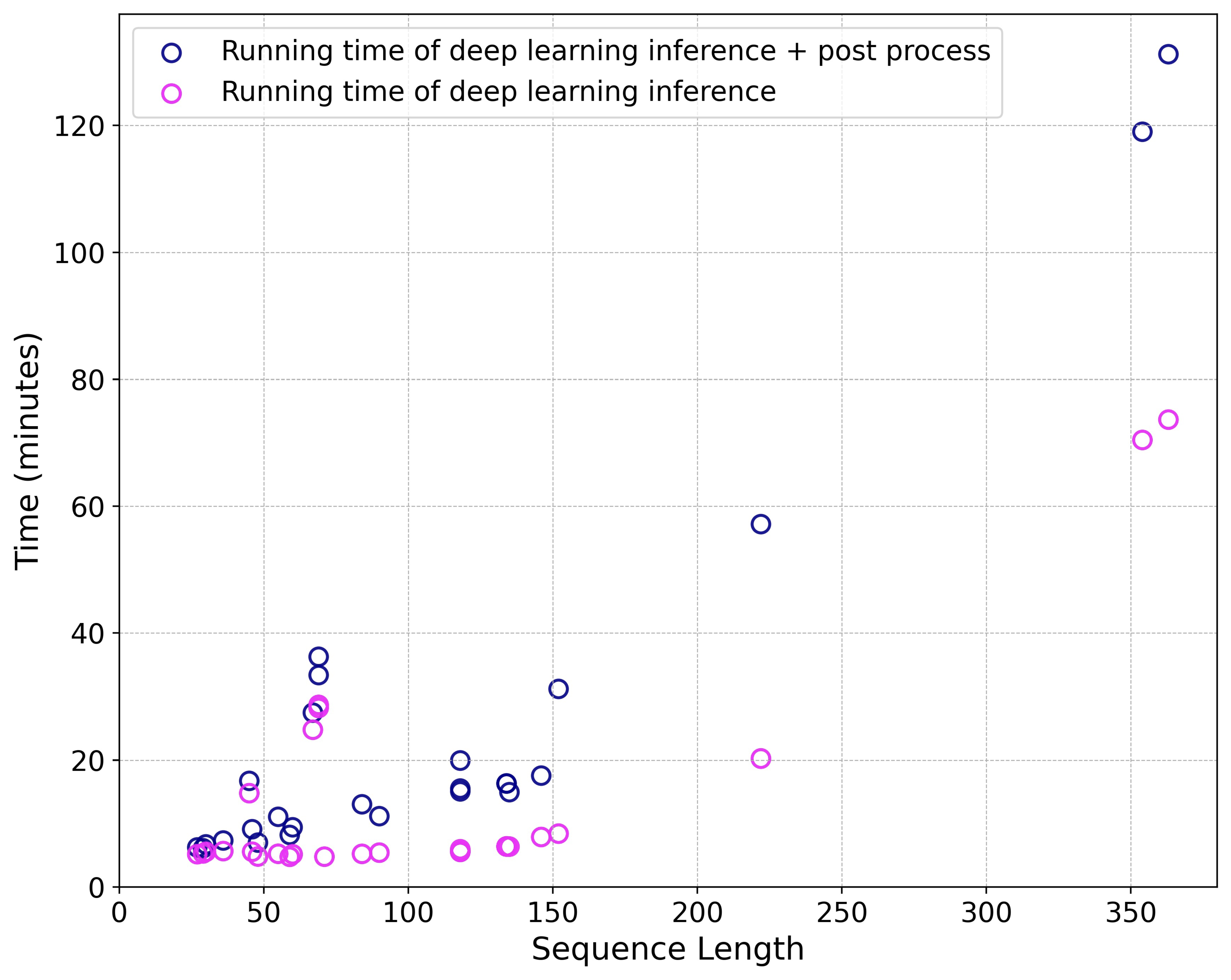

Supplement: S9 Fig — Underlying numerical data for this figure can be found in S1 Data (see sheets “S1_Data_S9”). (TIF) [file pbio.3003659.s014.tif]

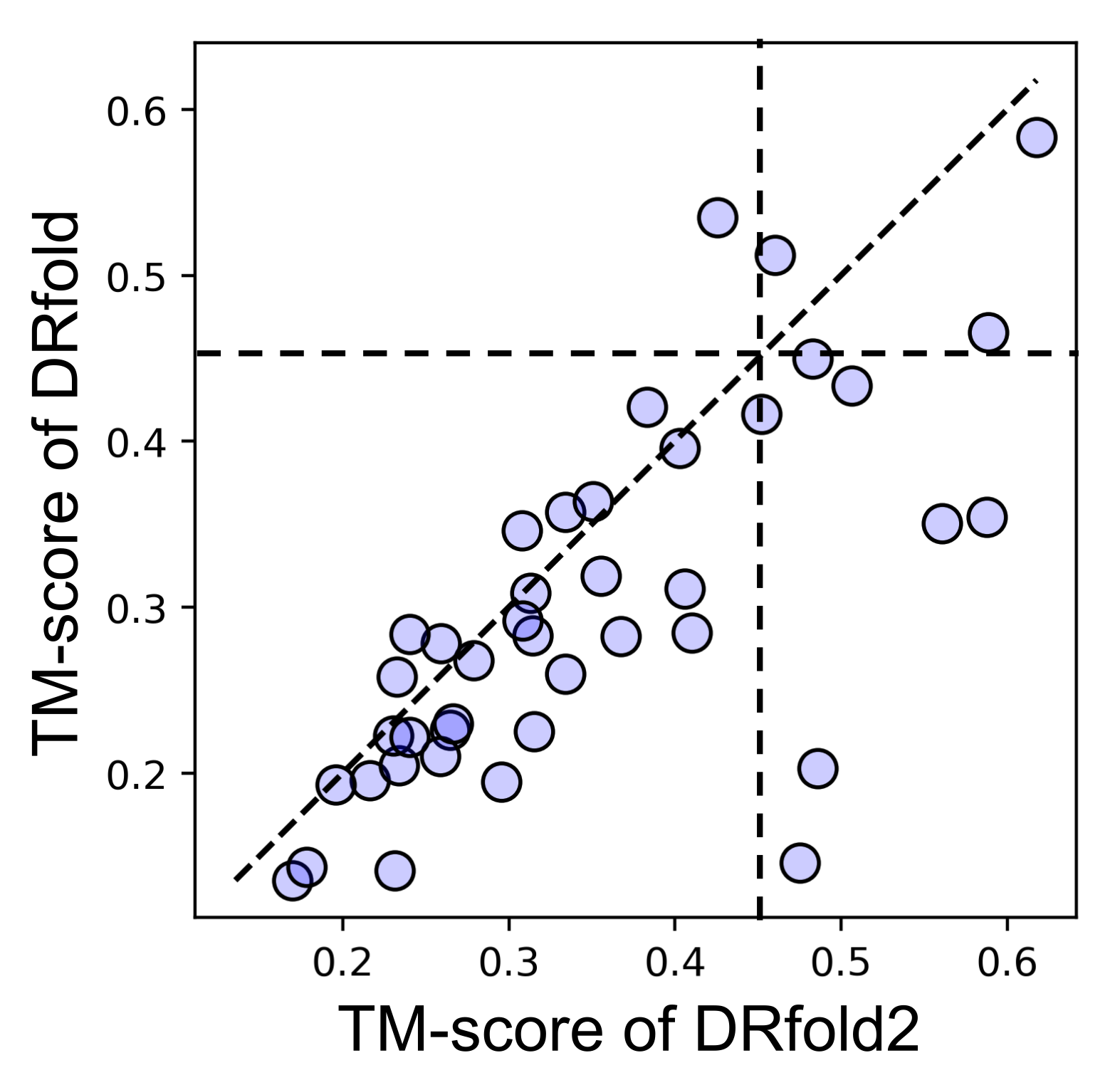

Supplement: S10 Fig — Underlying numerical data for this figure can be found in S1 Data (see sheets “S1_Data_S10”). (TIF) [file pbio.3003659.s015.tif]

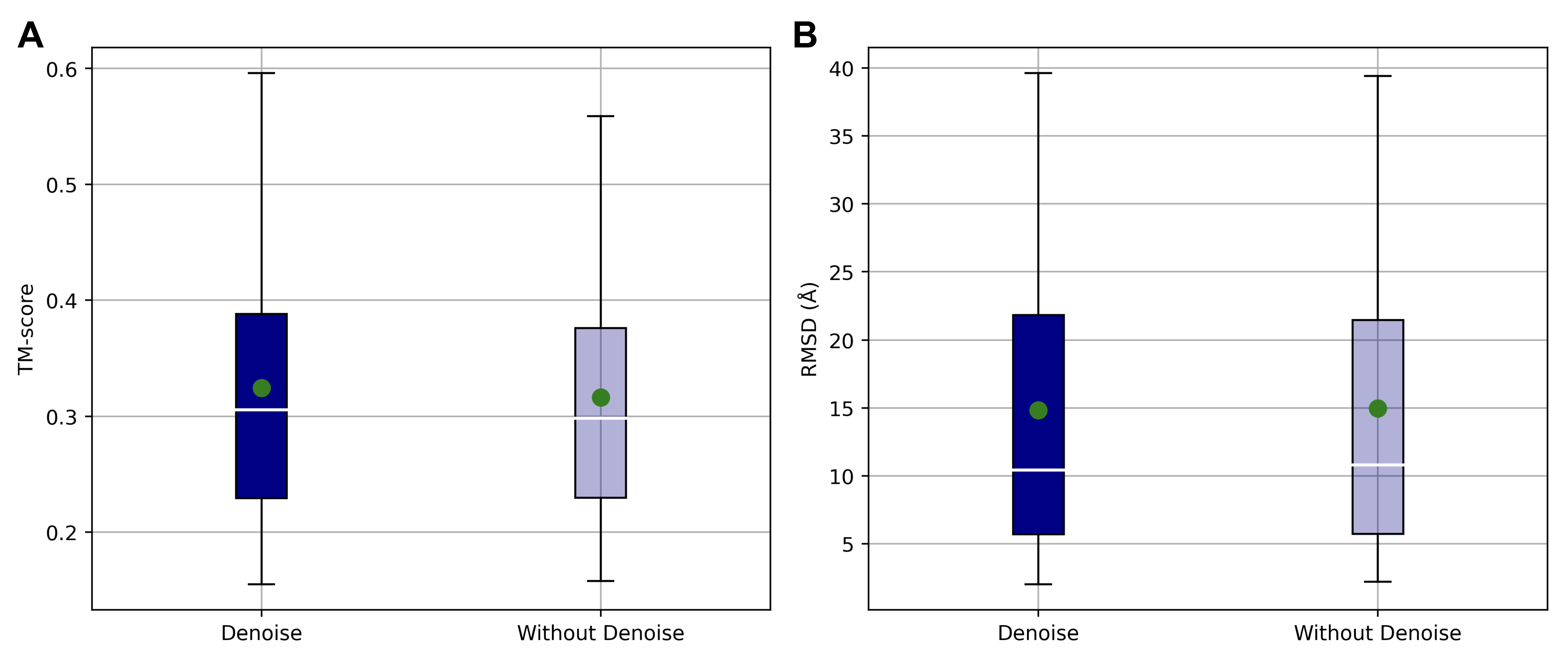

Supplement: S11 Fig — (A) TM-score comparison (0.324 with denoising versus 0.316 without). (B) RMSD comparison (14.827 with denoising versus 14.927 without). Green points denote the mean values, and white horizontal lines mark the medians. Underlying numerical data for this figure can be found in S1 Data (see sheets “S1_Data_S11”). (TIF) [file pbio.3003659.s016.tif]

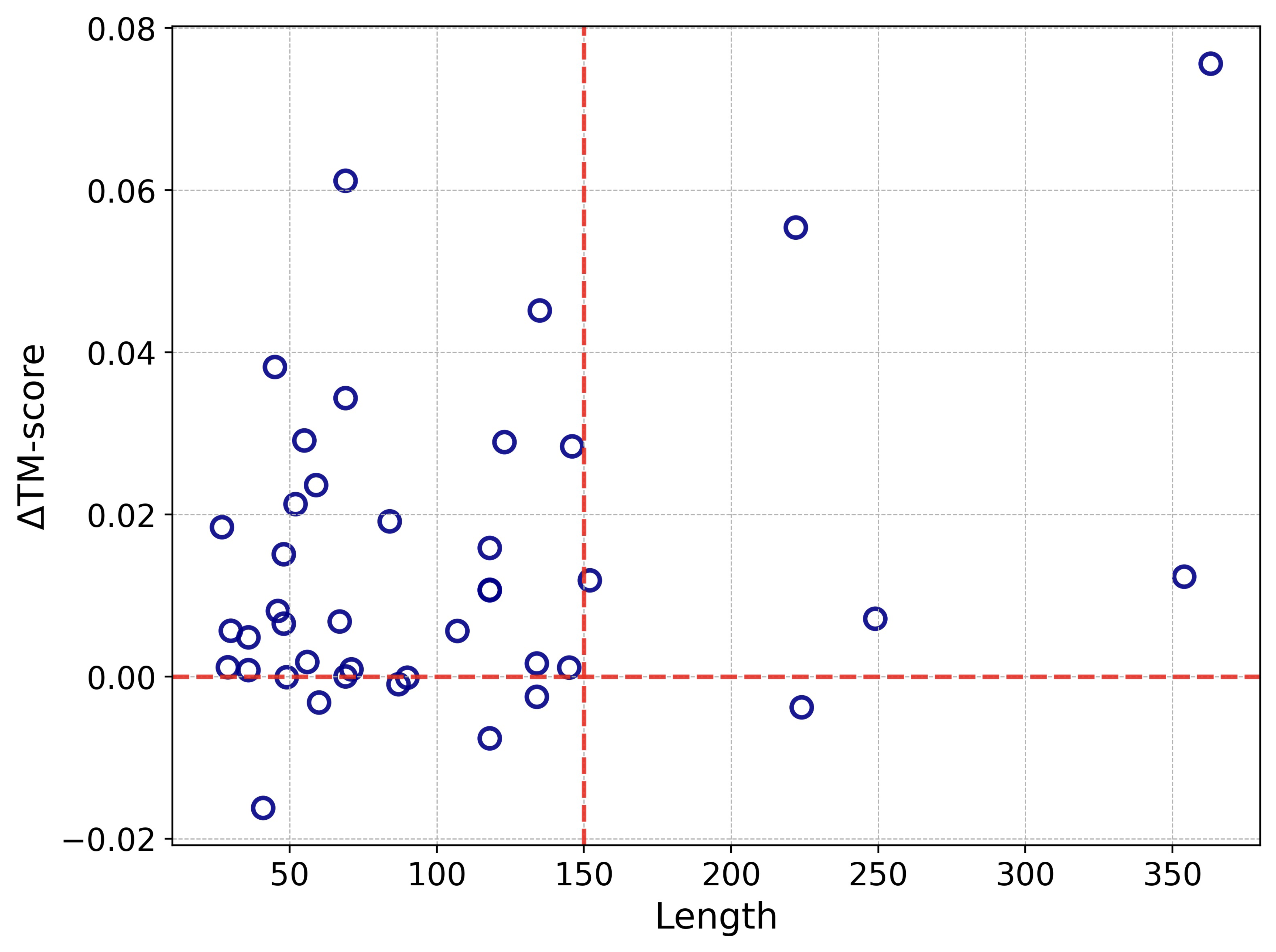

Supplement: S12 Fig — Underlying numerical data for this figure can be found in S1 Data (see sheets “S1_Data_S12”). (TIF) [file pbio.3003659.s017.tif]
