# Supplementary material for: DRfold2 is a deep learning-based tool that enables efficient and accurate RNA structure prediction
Source: PLoS Biol. 2026 Feb 17;24(2):e3003659. doi: 10.1371/journal.pbio.3003659 (PMC12931758; doi:10.1371/journal.pbio.3003659)
Supplement: Attachment — Submitted filename: Response to Reviewers.docx [file pbio.3003659.s021.docx]

**Response to Reviewer #1**

We very much appreciate the thoughtful, constructive comments from the Reviewer, which we found highly valuable for improving the quality and clarity of the DRfold2 manuscript. The major concerns raised primarily focus on the need for additional structural quality assessments (geometry, topology, torsion-angle similarity), and consistency between coarse-grained and full-atom models. In the revision, we have performed the requested analyses, added new evaluation metrics, expanded relevant discussions, and corrected all minor issues, with all changes highlighted in yellow.

1. **The Reviewer commented:**

***Li et al. introduce DRfold2, a deep learning model for ab initio prediction of 3D structures of single stranded RNAs, building on its predecessor, DRfold. A key innovation of DRfold2 is the integration of the RNA Composite Language Model (RCLM), which captures rich, sequence-dependent contextual features and significantly enhances structural modeling performance. DRfold2 demonstrates high accuracy compared to existing methods across diverse benchmarks, including RNA-Puzzles and CASP, with notable improvements in modeling complex and large RNA molecules. The paper is clear and extremely well illustrated. However, I have several questions and comments to the authors, that I would like them to address.***

We appreciate the summery and positive comments from the Reviewer on the work.

2. **The Reviewer commented:**

***(1) Recently, several studies have emerged in which RNA-Puzzles/CASP assessors evaluated the quality of RNA structure predictions not only based on their similarity to reference structures but also considering the geometry and topology of the predicted structural models. For example, in the study by Carrascoza et al., 2022, titled "Evaluation of the stereochemical quality of predicted RNA 3D models in the RNA-Puzzles submissions," the authors pointed out that many models exhibit incorrect geometry (bond lengths, planarity, bond angles). Meanwhile, Gren et al., 2024, in their work "Knotted artifacts in predicted 3D RNA structures," examined the predicted models for topological irregularities (entanglements of structural elements and topological knots). I believe that post-prediction analysis of geometry and topology should become a standard and good practice among predictors and authors of new prediction tools. Therefore, I strongly suggest performing such analyses using available tools, such as MolProbity (for geometry) and RNAspider (for topology).***

Thank you for raising this important point. In the revised manuscript, we have updated the refinement program, performed both geometric and topological evaluations respectively. The results have been incorporated into a new section to assessing physical stereochemical quality and structural topology.

First, we update the description about the refinement in Section ***Structure optimization through CSOR protocol*** (Page 19-20):

Eventually, up to 5 models will be obtained, and each of them will be **refined** by RNArefine. Here, RNArefine (available at https://zhanggroup.org/RNArefine/) is a new protocol which first infers base-pairing and base-stacking interactions from the initial structure. Built on these interactions and a physics-based force field, it then performs atomic-level refinement through a hybrid Monte Carlo sampling procedure followed by L-BFGS energy minimization, iteratively improving both local geometry and global structural consistency.

A separate section, titled ***DRfold2 produces physically realistic RNA structures***, has been added accordingly (Page 7):

***DRfold2 produces physically realistic RNA structures***

In addition to achieving high global structure accuracy, DRfold2 also produces physically correct models, addressing a crucial problem in current RNA bioinformatics [38]. DRfold2 employs a hybrid refinement strategy that integrates Monte Carlo simulation with L-BFGS optimization to generate the final full-atomic models. We evaluated the geometric quality of the refined structures on the test set using three standard metrics, i.e., the root-mean-square (RMSs) of bond length and bond angle deviations, and the clash score (**S3 Table).** The corresponding values for native structures are also provided as references. The refinement procedure substantially improves all three metrics, reducing them from 0.042, 5.854, and 42.823, to 0.012, 1.559, and 2.171, respectively. Notably, DRfold2’s refinement step yields structures with an average clash score even lower than that of the experimental structures, highlighting its strong physical regularization capability.

**S3 Table** further reports the number of structures containing at least one entanglement [39], as identified by RNAspider, a webserver for detecting topological entanglements in RNA 3D structures [40]. Interestingly, although DRfold2 refinement is not explicitly designed to resolve such motifs, the refinement successfully removes one D&L interlace, in which a dinucleotide step (D) and a loop (L) are interwoven (**S3 Fig**).

.

**S3 Table. Physical geometry indexes of the first models by DRfold2 and experimental structurers.**

|  | RMS_bonds_ | RMS_angles_ | Clash score | Number of Entanglements |
| --- | --- | --- | --- | --- |
| DRfold2  (without refinement) | 0.042 | 5.854 | 42.823 | 3/41 |
| DRfold2 | 0.012 | 1.559 | 2.171 | 2/41 |
| Experimental  structure | 0.004 | 0.778 | 4.205 | 0/41 |

**S3 Fig. Structure visualization of the predicted conformations of target 8vci_A generated by DRfold2 before and after refinement. (A)** Prediction without refinement. **(B)** Prediction after refinement. Red dots indicate D&L interlace points. The structural visualizations are screenshots obtained from the RNAspider web server.

**New References**

38. Carrascoza F, Antczak M, Miao Z, Westhof E, Szachniuk M. Evaluation of the stereochemical quality of predicted RNA 3D models in the RNA-Puzzles submissions. Rna. 2022;28(2):250-62.

39. Gren BA, Antczak M, Zok T, Sulkowska JI, Szachniuk M. Knotted artifacts in predicted 3D RNA structures. PLOS Computational Biology. 2024;20(6):e1011959. doi: 10.1371/journal.pcbi.1011959.

40. Luwanski K, Hlushchenko V, Popenda M, Zok T, Sarzynska J, Martsich D, et al. RNAspider: a webserver to analyze entanglements in RNA 3D structures. Nucleic Acids Research. 2022;50(W1):W663-W9. doi: 10.1093/nar/gkac218.

3. **The Reviewer commented:**

***(2) An interesting metric used in RNA-Puzzles (see publications related to successive rounds of RNA-Puzzles) is the angular MCQ measure, which does not require structural alignment and reflects similarity in torsion angle space. Torsion angles are widely used in the analysis and determination of structures based on NMR data; also an RNA language model has been developed that is based specifically on torsion angles. I suggest the authors added a torsion angle-based metric to the set of evaluation measures they use for assessing predictions. Examples of easyto-use implementations of such metrics can be found, among others, in the RNAtango web service.***

Thank you for the constructive comments. We have added the MCQ torsion-angle metric following the Reviewer’s recommendation and incorporated the analysis into the revised manuscript. The metric allows us to evaluate the local geometric accuracy of DRfold2, complementing the global and stereochemical assessments.

We have added the following contents as the last two paragraphs of Section ***DRfold2 produces physically realistic RNA structures*** (Page 7):

We further examined the local torsion-angle similarity by evaluating the Mean of Circular Quantities (MCQ), a torsion-based distance metric that assesses the local geometry defined by every consecutive four atoms, with a lower MCQ value indicating better local structure [41]. **S4 Fig** presents the MCQ comparison across all baseline methods and DRfold2. On average, DRfold2 achieves an MCQ of 23.8, significantly outperforming many control methods (RNAComposer, trRosettaRNA, and RhoFold). For example, RNAComposer, which assembles structures using a high-quality fragment library and thus can generally provide good local torsion geometry, still shows a higher MCQ of 26.1 (p-value = 1.06E-03, one-tailed t-test). The MCQ value of DRFold2 is, however, comparable or slightly higher than the rest of the control methods (RoseTTAFoldNA, DeepFoldRNA, AlphaFold3), suggesting potential future improvements, for example, through the incorporation of torsion-based energy potential.

In summary, DRfold2 provides not only accurate global folds but also physically well-behaved structures, ensuring that the predicted models are suitable for reliable downstream experimental design and computational analyses.

.

**S4 Fig. Box plots comparing of MCQ of control methods and DRfold2 respectively.** Green points indicate means and white horizontal lines show medians.

**New References**

41. Zok T, Popenda M, Szachniuk M. MCQ4Structures to compute similarity of molecule structures. Central European Journal of Operations Research. 2014;22(3):457-73. doi: 10.1007/s10100-013-0296-5.

4. **The Reviewer commented:**

***(3) The TM-score values for the obtained predictions are poor. In the protein field, it is generally accepted that a TM-score < 0.5 indicates negligible similarity between the model and the target and is equivalent to a random prediction. TM-score values around 0.3 are therefore not evidence of correct or accurate predictions - quite the opposite - they indicate that the predictions are inaccurate. How do the authors comment on this fact?***

We thank the Reviewer for raising this question. Although the absolute TM-scores are modest, our analysis shows that DRfold2 can still reliably capture meaningful global structural patterns for the majority of RNA targets, better than random expectation. Specifically, DRfold2 shows consistently low failure rates across different sequence identity thresholds and correctly predicts the overall fold for many targets, outperforming established control methods.

Accordingly, we have added the following analysis and discussion as the last paragraph in Section ***DRfold2 outperforms existing methods in global RNA tertiary structure prediction*** (Page 6)

We acknowledge that the highest TM-score among all methods, including DRfold2, is relatively low (0.368, achieved by DRfold2 combined with AlphaFold3 potentials), which may raise concerns about whether these methods can reliably predict RNA structures. We thus analyzed the number of targets according to two TM-score thresholds, 0.21 and 0.45. The lower threshold (0.21) corresponds to the expected normalized TM-score between any two random structures, while the higher threshold (0.45) indicates that two conformations belong to the same fold [32]. Using these criteria, DRfold2 successfully predicts 10 targets with the correct structural fold (TM-score ≥ 0.45) and only 3 targets below the random threshold (TM-score < 0.21). This performance is slightly better than AlphaFold3 predictions (8 and 3, respectively) and largely outperforms the non-deep learning method, RNAComposer, which fails to predict any correct folds and produces 13 structures worse than random. Considering the very low failure rate (TM-score < 0.21) of DRfold2 across different sequence identity thresholds, i.e., 7.31%, 8.11%, 14.29% and 11.11% for identity thresholds of 0.8 to 0.5, respectively, we conclude that despite the modest absolute TM-scores, DRfold2 can reliably capture meaningful overall structural topology for the majority of RNA targets, even in cases of low sequence homology.

5. **The Reviewer commented:**

***(4) The evaluation of the predicted models is very coarse-grained if it reduces the entire nucleotide to a single representative atom. The same value of a given metric is interpreted very differently when calculated for a full-atom structure compared to a coarse-grained one. For example, a value indicating a rather poor-quality coarse-grained model may actually be quite good for a full-atom model, especially if the metric is distance-based and the evaluation involves large molecules. Applying the same scoring function to both full-atom and coarse-grained models yields completely different metric values. These values are not directly comparable. Why don't the authors assess the similarity using full-atom structures?***

We thank the Reviewer for raising this important point. We agree that coarse-grained and full-atom evaluations may capture different aspects of structural accuracy, and that relying solely on coarse-grained metrics may overlook certain geometric or stereochemical details. In response, we have expanded our analysis to include full-atom accuracy measures (in addition to **Responses 2&3**) and clarified the rationale for using coarse-grained metrics as the primary metric.

To address this concern, we first performed full-atom RMSD evaluations across all methods, as described in 8^th^ paragraph of Section ***DRfold2 outperforms existing methods in global RNA tertiary structure prediction*** (Page 5-6):

More strictly, we further analyzed the full-atom RMSD of control methods and DRfold2 (**S1 Fig**). DRfold2 consistently outperforms all control methods, achieving an average full-atom RMSD of 14.026 Å. Incorporating the AlphaFold3 potential further reduces the full-atom RMSD to 13.767 Å, highlighting the robustness of DRfold2 in generating accurate all-atom conformations.

**S1 Fig. Box plots comparing of full-atom RMSD of control methods and DRfold2 respectively.** Green points indicate means and white horizontal lines show medians.

Moreover, we have added a discussion on the structure evaluation as the second paragraph in Section ***Discussion*** (Page 13):

Although the performance of DRfold2 is primarily evaluated using coarse-grained metrics such as TM-score, we have also assessed the predicted structures using full-atom RMSD, and DRfold2 consistently outperforms all control methods. This indicates that the global topology captured by coarse-grained metrics is also well reflected at the full-atom level. While DRfold2 does not achieve the best performance for certain full-atom descriptor (MCQ), the MCQ metric, however, marginalizes local variations and assumes that all local regions contribute equally. In reality, local deviations in critical loops or junctions can have a larger impact on the overall global topology, and may not be fully captured by averaged local metrics. Other full-atom physical validation metrics, such as RMS deviations of bonds and angles or clash scores, focus primarily on geometric plausibility and the likelihood of the structure itself, but do not specifically evaluate $P(structure|sequence)$, i.e., whether the predicted conformation is compatible with the RNA sequence. Well-established coarse-grained global metrics, such as TM-score and RMSD, while sometimes simpler, better reflect this sequence-informed structural prediction accuracy. Nevertheless, by combining coarse-grained global metrics with selective full-atom evaluations, our evaluation approach confirms that DRfold2 predictions are both globally accurate and physically realistic.

**6. The Reviewer commented:**

***(5) Typos and minor issues:***

***- "likelihood[26]" (no space between)***

***- "assumption, First" -> "assumption, first"***

We thank the Reviewer for pointing out these typographical issues. Both errors have been corrected in the revised manuscript.

**Response to Reviewer #2**

We thank the Reviewer for the insightful and constructive comments. The Reviewer’s major concerns are on the benchmarking and methodological details. In response, we have revised the manuscript to improve benchmarking setups and methodology descriptions. Below, we provide point-by-point responses to all comments, with all changes highlighted in yellow.

1. **The Reviewer commented:**

***Reviewer #2: This paper introduces DRfold2, a deep learning framework for the single-sequence RNA 3D structure prediction. They introduce two things: first, a pre-trained RNA Composite Language Model (RCLM) that utilizes a composite likelihood objective to capture pairwise nucleotide interactions from sequence data alone more effectively; and second, a Denoising RNA Structure Module (DRSM) integrated into the pipeline. The authors conduct extensive benchmarks against several state-of-the-art methods on a non-redundant test set.***

We appreciate the nice summary on the work by the Reviewer.

2. **The Reviewer commented:**

***Despite the claimed strengths, the paper has several weaknesses that should be addressed.***

***1. AlphaFold3 is one of the current state-of-the-art methods, and its exclusion from the main comparative figures (e.g., Figure 2) makes it difficult for the reader to immediately contextualize DRfold2's performance. While the dedicated analysis in Figure 6 is valuable, integrating AlphaFold3 into the primary benchmark plots would provide more context for DRfold2 against its top competitor.***

We thank the Reviewer for this valuable suggestion. In the revised manuscript, we have incorporated AlphaFold3 directly into the main benchmarking.

Specifically, we have moved the comparison between DRfold2 and AlphaFold3 into the main comparison section ***DRfold2 outperforms existing methods in global RNA tertiary structure prediction***. the following contents are added to the first paragraph of the section (Page 3):

We also compare DRfold2 with the latest iteration of AlphaFold, AlphaFold3 [31], for RNA 3D structure prediction. For doing this, we submitted the RNA sequences in our test set to the AlphaFold Server and obtained the predicted structures of AlphaFold3 with default seed configurations.

We have also revised the second paragraph of this section (Page 4) to incorporate the performance of AlphaFold3 together with other control methods. The updated text now reads:

Within the test dataset, there are 9, 21, 37, and 41 targets with sequence identities below 50%, 60%, 70%, and 80%, respectively, relative to the training dataset. In general, prediction accuracy of DRfold2 increases for the targets with higher sequence identity to the training set. Notably, this trend holds for all control methods even though no filters was applied to their training dataset, probably because these methods were trained on RNAs released before 2024 which have a similar homologous distance to the new test dataset used in this study. It is observed that DRfold2 consistently achieves the highest average TM-score among all methods across all sequence identity thresholds. For example, at the 80% cutoff, DRfold2 attains an average TM-score of 0.350 which is 19.9% higher than DeepFoldRNA (TM-score=0.292). The difference is statistically significant, with a p-value of 6.37E-04 in the one-tailed Student’s t-test, despite the low degree of freedom, i.e., the limited sample size of 41. Even with the most stringent test subset containing 9 test targets (50% sequence identity cutoff), DRfold2 achieves an average TM-score of 0.275, which is 20.0% higher than RoseTTAFoldNA (TM-score=0.229), with a p-value of 1.43E-02 (one-tailed t-test). Meanwhile, DRfold2 exhibits a lower average RMSD than all other control methods across different sequence identity thresholds. DRfold2 also achieves a slightly higher TM-score (0.350) and lower RMSD (14.40 Å) than AlphaFold3 (0.347 and 16.00 Å) (**S1 Table**); however, the difference between the two is not statistically significant, with p-values of 8.91E-01 and 1.37E-01 for TM-score and RMSD respectively, in two-tailed Student’s t-test.

We have updated **Fig 2** and **Fig 3** to include both AlphaFold3 and the hybrid DRfold2+AlphaFold3 potential (denoted DRAF). The corresponding descriptions are 4^th^ and 5-7^th^ paragraphs, respectively, in Section ***DRfold2 outperforms existing methods in global RNA tertiary structure prediction***:

4^th^ paragraph for Fig 2:

In **Fig 2C**, we present one example of a CPEB3 HDV-like ribozyme from Chimpanzee (PDB ID: 7QR3), a 69-nucleotide RNA molecule, where DRfold2 shows advantage in modeling tertiary structure. Among the four better-performing methods in our benchmark, DRfold2 accurately captures the topology of the ribozyme with a TM-score and RMSD of 0.588 and 2.72 Å, respectively. In contrast, DeepFoldRNA and AlphaFold3, while capturing the general helical arrangement, show notable deviations in the orientation of the hairpin loop. As a result, the RMSD becomes 5.68 Å and 7.98 Å respectively, which is at least twice the deviation of DRfold2. RhoFold shows errors in predicting the spatial organization of the junction regions, with TM-scores reducing to 0.323. Note that the maximum sequence identity between this target and the training RNAs is only 60.9%, demonstrating that the DRfold2 pipeline can provide reasonable structure predictions even for novel sequences that are not homologous to the training data.

5-7^th^ paragraphs for Fig 3:

**Figs 3A&B** provide head-to-head TM-score and RMSD comparisons by the DRfold2 and AlphaFold3. Although DRfold2 outperforms AlphaFold3 in several low- to medium-quality models, AlphaFold3 excels in two targets with TM-score above 0.6. A detailed comparison between DRfold2 and AlphaFold3 across different target annotations is shown in **S2 Table**, where pseudoknots were annotated using RNApdbee [34] and all other categories follow the official NAKB annotations [35]. DRfold2 achieves a higher average TM-score on targets containing pseudoknots, suggesting that it produces more accurate global folds in these cases. However, in terms of pseudoknot recovery rate, DRfold2 is notably lower than AlphaFold3, i.e., 56.3% versus 79.1%, indicating that AlphaFold3 captures atomic-level base-pairing conformations more accurately. For reference, other control methods have substantially lower pseudoknot recovery rates, i.e., 19.0% and 2.0% for RoseTTAFoldNA and RhoFold, respectively. For the remaining NAKB-defined categories, DRfold2 performs better on Aptamer, Catalytic, and G-quadruplex RNAs, whereas AlphaFold3 shows advantages on Virus and Riboswitch targets.

While most of our above discussions focus on the top-rank models by DRfold2, we have identified several targets for which DRfold2 generated correct structures, but the post-process step failed to select them as the first-rank models. **Fig 3C** illustrates one such examples from the coxsackievirus B3 cloverleaf RNA (PDB ID: 8DP3), which contains 90 residues forming a 4-way junction fold [36]. By checking the first-rank models, both DRfold2 and AlphaFold3 failed to correctly predict the direction of the 4-way junction, with RMSDs to the native around 20 Å. To further explore AlphaFold3's capabilities, we submitted the same sequence to AlphaFold Server 20 times with different seeds, resulting in 100 predicted conformations. The middle panel of **Fig 3C** shows the best conformation with the highest TM-score, which still exhibits a poor RMSD of 21.83 Å due to incorrect chain winding topology across the 4-way junction. In contrast, the 5^th^-ranked model from DRfold2 successfully predicted the junction’s direction, reducing the RMSD from 22.05 Å (first DRfold2 model) to 6.54 Å (right panel of **Fig 3C**). While this example highlights DRfold2’s capability to generate diverse and alternative conformations, it also underscores the need to improve the post-processing CSOR protocol for more effectively selecting and ranking the correct models from the DRfold2 decoy pool, e.g., through the combination of third-party algorithms.

Although DRfold2 and AlphaFold3 exhibit similar overall performance, the results in **Figs 3A-B** highlight the strong complementarity between the two programs, particularly in cases where predictions deviate from the diagonal line. This complementary behavior, along with the identified need to improve DRfold2’s model selection strategy, motivated us to explore whether combining the two approaches could lead to enhanced structure predictions. The optimization module in DRfold2 readily facilitates this integration by incorporating AlphaFold3 conformations as an additional potential term. Guided with this combined potential function, the optimized structures achieve higher quality models than those generated by either method alone, as shown in **Fig 2** labelled as DRAF, and in the detailed head-to-head comparison in **Figs 3D&E**. The hybrid approach achieves an average TM-score of 0.368 and RMSD of 14.02 Å, both being significantly better than that of AlphaFold3 (0.347 and 16.00 Å, with p-value = 4.86E-02 for TM-score and 3.16E-03 for RMSD, one-tailed t-test). These results not only validate our hypothesis regarding the complementarity between DRfold2 and AlphaFold3 but also demonstrate the flexibility and extensibility of the CSOR module, enabling the seamless integration of external model features to further improve structure prediction in DRfold2.

.

**Fig 2. RNA structure prediction results on the test dataset by different methods across different sequence identity thresholds. (A)** Box plots of TM-score and RMSD for eight RNA structure prediction methods evaluated at different sequence identity cutoffs (50-80%) between training and test datasets, with green point and white horizontal line representing the mean and median values respectively. **(B)** Cumulative distribution plots of TM-score and RMSD for the predicted models. Upper panels display the fraction of models above different TM-score thresholds, while lower panels show the fraction of models below different RMSD values. **(C)** A representative modeling example from Chimpanzee CPEB3 HDV-like ribozyme (PDB ID: 7QR3), with models predicted by four better-performing methods (blue cartoons) overlaid on experimental structure (gray cartoons).

**Fig 3. Comparative analysis of DRfold2 and AlphaFold3 for RNA structure predictions. (A-B)** Head-to-head TM-score and RMSD comparisons between DRfold2 and AlphaFold3. **(C)** Structural visualization of the example from coxsackievirus B3 cloverleaf RNA (PDBID: 8DP3), showing experimental structure, AlphaFold3's best prediction from 100 models, and 5^th^ model of DRfold2, respectively. Structures are rainbow-colored from 5' (blue) to 3' (red) end. **(D-E)** TM-score and RMSD comparisons between AlphaFold3 and DRfold2 with DRfold2+AlphaFold3 potential. **(F)** Performance comparisons between DRfold2 and Alphafold3 grouped by MSA depth retrieved by AlphaFold3. Green points indicate means and white horizontal lines show medians.

3. **The Reviewer commented:**

***22. The paper's central claim of being a "single-sequence" method is not fully leveraged or validated against MSAbased approaches in their domain of weakness. The key advantage of a single-sequence model should be its robust performance on targets with few or no homologs. The study would be much more compelling if it included an analysis comparing DRfold2 against MSA-dependent methods (like trRosettaRNA or an MSA-enabled AlphaFold3) on targets binned by their MSA depth. Such an experiment would directly test and highlight the practical utility of the single-sequence approach in the very scenarios where it is most needed.***

We thank the Reviewer for this insightful suggestion. In the revision, we have added a dedicated analysis that evaluates DRfold2, AlphaFold3 (with and without MSA), and the hybrid method (DRAF) across targets binned by MSA depth. We find that DRfold2 performs better on RNA targets with few or no detectable homologues.

Accordingly, we have added a new result Section ***DRfold2 enables robust RNA structure prediction with few homologu*es**:

***DRfold2 enables robust RNA structure prediction with few homologues***

Next we examine how the performance of DRfold2 varies across different levels of evolutionary information, as reflected by the MSA depth retrieved by AlphaFold3. In **Fig 3F,** we present this comparison together with AlphaFold3. For reference, we also include the performance of DRAF as well as that of AlphaFold3 without MSA input. In general, both DRfold2 and AlphaFold3 achieve better performance with the increase of numbers of homologous sequences, likely due to richer co-evolutionary constraints. When no MSA is available, AlphaFold3 achieves an average TM-score of only 0.333, which is moderately lower than its full-version performance (0.347).

Although DRfold2 and AlphaFold3 show comparable overall accuracy, their behavior differs across MSA-depth groups. For targets without any detectable homologs (MSA depth = 0), DRfold2 achieves slightly higher accuracy (TM-score = 0.300) compared to AlphaFold3 (TM-score = 0.287). A similar trend is observed for low-homology targets (MSA depth 1–10), where DRfold2 outperforms AlphaFold3 with TM-scores of 0.442 versus 0.407. For targets with richer evolutionary information (for example, MSA depth 11–20), however, AlphaFold3 performs better (TM-score = 0.357 versus 0.311 for DRfold2). Nonetheless, the hybrid folding pipeline, DRAF, which integrates DRfold2 predictions with the AlphaFold3 potential, achieves the highest accuracy (TM-score = 0.368) in this group, demonstrating the complementarity of the two approaches.

Taking together, these results further highlight the complementary strengths of DRfold2 and AlphaFold3. DRfold2 is trained primarily on single-sequence features enriched with co-evolutionary signals derived from RCLM. The hidden universal covariance learned by RCLM becomes more valuable, for example, when explicit homologues are insufficient or not detected. Consequently, DRfold2 can provide more reliable predictions for RNAs with few or even no homologues.

**Fig 3. Comparative analysis of DRfold2 and AlphaFold3 for RNA structure predictions. (A-B)** Head-to-head TM-score and RMSD comparisons between DRfold2 and AlphaFold3. **(C)** Structural visualization of the example from coxsackievirus B3 cloverleaf RNA (PDBID: 8DP3), showing experimental structure, AlphaFold3's best prediction from 100 models, and 5^th^ model of DRfold2, respectively. Structures are rainbow-colored from 5' (blue) to 3' (red) end. **(D-E)** TM-score and RMSD comparisons between AlphaFold3 and DRfold2 with DRfold2+AlphaFold3 potential. **(F)** Performance comparisons between DRfold2 and Alphafold3 grouped by MSA depth retrieved by AlphaFold3. Green points indicate means and white horizontal lines show medians.

4. **The Reviewer commented:**

***3. The contribution and rationale of the Denoising RNA Structure Module (DRSM) is not sufficiently justified. The paper lacks an ablation study to quantify the performance gain specifically attributable to this module. For instance, what is the accuracy of the model without the noise injection during training? Furthermore, the term "denoising" is misleading, as the method adds noise to the initial state (identity rotations and zero-translation vectors) and deterministically predicts the structure, rather than the common approach to iteratively remove noise from corrupted ground-truth structures. A more detailed explanation of why this noise-conditioned training is advantageous over simply learning the transformation from a zero-initialized state, as in AlphaFold2, is needed to fully motivate its design.***

We thank the Reviewer for pointing out the ambiguity in our original description. We performed an ablation study by retraining a model without the denoising procedure. This retrained baseline showed a slight decrease in performance.

Regarding the rationale for using denoising, our design is inspired by the denoising autoencoder framework rather than diffusion models. We believe this strategy improves robustness, especially given that RNA local nucleotide conformations defined by phosphate P, C4′, and glycosidic N atoms, are more flexible than protein backbone coordinates that AlphaFold2 mainly predicts.

In the revised manuscript, we have clarified the rationale of DRSM, added an ablation study, and explained why noise-conditioned training benefits structure prediction with examples. The added text is the second paragraph of Section ***End-to-end RNA structure prediction with the Denoising Structure Module***:

The parameters *w* and *ε* were chosen conservatively to introduce minor perturbations to the input without destroying the structural signal. The module is inspired by the denoising autoencoder [55] principle, i.e., DRSM will learn to map a distribution of perturbed initial conformations to the correct structure, rather than relying on a single fixed initialization as in AlphaFold2. This noise-conditioned training expands the diversity of the input space and consequently improves generalization. Such improvement is supported by the ablation (no-noise baseline), which shows a minor decrease in TM-score (**S11 Fig**). A related idea is also observed in AlphaFold3 [31] in the ‘ref_pos’ feature, in which the reference conformer is randomly rotated and translated during training. This further supports the effectiveness of introducing stochastic perturbations into the initial structural states.

**S11 Fig. TM-score and RMSD comparisons between models trained with and without the denoising strategy.** (**A)** TM-score comparison (0.324 with denoising versus 0.316 without). **(B)** RMSD comparison (14.827 with denoising versus 14.927 without). Green points denote the mean values, and white horizontal lines mark the medians.

**New references**

31.	Abramson J, Adler J, Dunger J, Evans R, Green T, Pritzel A, et al. Accurate structure prediction of biomolecular interactions with AlphaFold 3. Nature. 2024;630(8016):493-500. doi: 10.1038/s41586-024-07487-w.

55.	Kramer MA. Autoassociative neural networks. Computers & Chemical Engineering. 1992;16(4):313-28. doi: https://doi.org/10.1016/0098-1354(92)80051-A.

5. **The Reviewer commented:**

***4. There is a notable omission of any quantitative data on computational efficiency. The title claims the method is "efficient," but without runtime information, particularly for involving the multi-decoy CSOR post-processing protocol. It is difficult for the research community to assess the method's practical inference time.***

We thank the Reviewer for highlighting this important point. In our manuscript, the term efficiency mainly refers to the parameter efficiency of RCLM compared with existing RNA language models.

To clarify this, we have added detailed comparisons of model sizes to better illustrate the efficiency with which RCLM learns physical interaction patterns. The following description has been added to section ***RCLM more effectively captures co-evolutionary patterns than existing language models*** (Page 10):

It is important to note that, RCLM, containing 47M parameters, demonstrates superior performance in unsupervised contact precision tasks, with only 50% of the model size of RNA-FM (100M) and RNAErnie (87M), and even less than 10% of RiNALMo (650M). Such efficiency makes this form of language model training a promising approach for RNA sequence language model learning. Our next step is to upscale this methodology to explore the full potential of RCLM.

We further highlight such efficiency in Section Discussion (Page 13-14):

The RCLM model achieves significantly higher unsupervised contact prediction precision compared to existing representative methods using only 10% of the parameters, demonstrating a superior ability and efficiency in capture co-evolutionary signals solely from sequence data.

As suggested by the Reviewer, we also added a detailed runtime analysis to make the inference and post-processing costs transparent to the research community. The new section reads: (Page 12-13)

***DRfold2 running time analysis***

The DRfold2 pipeline can be broadly divided into two stages: (1) deep learning inference for generating multiple decoys and geometry restraints, and (2) post-processing to obtain the final structure. In S9 Fig, we present a running time analysis of both the deep learning inference and the post-processing against RNA length. To avoid bias, the refinement step using RNArefine is not included in this analysis, as its runtime is fixed at 180 minutes.

We observe that for the majority of targets, DRfold2 can generate 80 coarse-grained conformations within 40 minutes, i.e., < 30 seconds per model on a single RTX 5000 GPU without parallelization. Even for the largest tested RNA, the maximum inference time remains <1 minute per conformation. The post-processing stage is also well controlled despite the included complex optimization procedures, and its running time is approximately 0.834× that of the deep learning inference.

In summary, for typical RNA sizes (RNA length < 200 nts), DRfold2 completes folding within 40 minutes, while larger RNAs of ~350 nucleotides can be predicted within <120 minutes to obtain an accurate global fold. An additional 180-minute refinement step can further produce high-quality full-atomic structures with largely improved physical correctness.

**S9 Fig. Running time analysis of deep learning inference and post-processing steps across RNA lengths.**

6. **The Reviewer commented:**

***5. The RCLM part does not provide any details on the configurations of RCLM like number of parameters or layer number while such details exist for other methods. For RCLM architecture, it is confusing that Fig1.C denotes a 'Outer Product Mean' process, while it should be something used for MSA. For RCLM training, it is mentioned that supervising second-order interactions can better approximate full RNA likelihood, but (x_i, x_j) and (x_j, x_i) are the same pair, meaning that the matrix-based likelihood prediction is supervised by a symmetric matrix. What is the point of incoming/outgoing triangle update operation here then?***

We thank the Reviewer for these detailed questions regarding the RCLM design and training. We have revised the manuscript to clarify all three aspects raised by the Reviewer, as detailed below.

(1) For the question regarding detailed configurations of RCLM, we have added the detailed configurations and model size of RCLM in Sections ***RNA sequence representation learning by Composite Language Model*** (Page 16) and ***RCLM more effectively captures co-evolutionary patterns than existing language models*** (Page 10) respectively:

The RCLM model is composed of 18 transformer blocks, with hidden dimensions of 512 for sequence representations and 128 for pairwise representations, resulting in a total of 47,495,200 trainable parameters. The model was trained on ~30M RNA sequences from the RNAcentral database [45] (Release 22) over 67,000 batches, with a batch size of 128. The entire training process took about 15 days using a single NVIDIA A40 GPU.

and

It is important to note that, RCLM, containing 47M parameters, demonstrates superior performance in unsupervised contact precision tasks, with only 50% of the model size of RNA-FM (100M) and RNAErnie (87M), and even less than 10% of RiNALMo (650M). Such efficiency makes this form of language model training a promising approach for RNA sequence language model learning. Our next step is to upscale this methodology to explore the full potential of RCLM.

(2) For the question regarding RCLM architecture, the Reviewer is correct that Outer Product Mean is designed for MSA representations. The reason we keep this component is that a single sequence can be considered as a special case of an MSA, where no homologous sequences are aligned. We have added the following explanation to Section ***RNA sequence representation learning by Composite Language Model*** (Page 15):

Here, the RNA Transformer Block used in DRfold2 is based on the Evoformer architecture from AlphaFold2, which was originally designed to model MSA data. In our setting, a single RNA sequence can be viewed as a degenerate MSA with no aligned homologs. Since the underlying block architecture remains unchanged, we preserve the original AlphaFold2 formulation (e.g., Outer Product Mean) for this component.

(3) For RCLM training. The Reviewer is correct that ${(x}_{i},x_{j})$ and ${(x}_{j},x_{i})$ represent the same pair. However, RCLM assigns them to different classes by considering the positional order of *i* and *j*. Thus, the pair labels are asymmetric. We explain this labeling scheme as follows in Section ***RNA sequence representation learning by Composite Language Model*** (Page 16):

In our setup, when position *i* and *j* are masked, while the pair labels ${(x}_{i},x_{j})$ and ${(x}_{j},x_{i})$ correspond to the same nucleotide pair, they are assigned to different classes based on the order of *i* and *j*. Specifically, the joint states of a nucleotide pair are categorized into 16 classes, and ${(x}_{i},x_{j})$ and ${(x}_{j},x_{i})$ fall into distinct (though related) categories. This symmetric and asymmetric operations in Sequence Embedder and RNA Transformer Blocks allow the model to capture directional or order-dependent correlations between nucleotide positions.

7. **The Reviewer commented:**

***5. 6. The difference between DRfold2 and DRfold should be clearly stated. The whole architecture looks similar despite the RCLM.***

We thank the Reviewer for raising this important point. In the revised manuscript, we now summarize the architectural and algorithmic differences between DRfold and DRfold2. While the overall pipeline structure appears similar, DRfold2 incorporates several upgrades that lead to significantly improved performance, as added in Section **Materials and methods**.

Additionally, the pipeline integrates a Denoising RNA Structural Module (DRSM), which employs a controlled perturbation strategy to robustly learn structural transformations by efficiently correcting noisy RNA conformations. The pipeline also features a carefully designed flexible end-to-end loss function with distance-dependent reweighting schema to help the model learn meaningful interaction patterns. Lastly, we extend the deep learning-based conformational pool for the proposed CSOR to identify the most promising structure and possible alternative conformations. More detailed technical comparisons of the newly introduced modules and algorithmic upgrades are summarized in **S5 Table**. Equipped with those advancements, DRfold2 significantly outperforms DRfold (**S10 Fig)** in TM-score (0.350 versus 0.300) with p-value of 8.03E-04 in one-tailed t-test, providing 2.5 times as many (from 4 to 10) foldable RNAs (TM-score > 0.45) on the test set used in this study.

**S5 Table. Detailed technical comparison between DRfold and DRfold2 across multiple categories.**

|  | DRfold | DRfold2 |
| --- | --- | --- |
| Input features | 1. Sequence feature 2. Predicted secondary structure | 1. Sequence feature 2. RCLM embedding |
| Training data | 1. PDB structures before Year 2021 | 1. Updated PDB structures before Year 2024 |
| Loss function | 1. FAPE loss 2. Distance loss | 1. Generalized FAPE loss 2. Distance loss |
| Robust strategies | 1. SVD orthogonalization | 1. SVD orthogonalization 2. Attention dropout 3. Denoising RNA Structure Module |
| Conformations | 1. 6 conformations | 1. 80 conformations |
| Post-processing | 1. Optimization 2. Refinement | 1. Clustering 2. Selection 3. Optimization 4. Refinement |
| Refinement program | 1. OpenMM | 1. RNArefine |

The definition of generalized FAPE (gFAPE) loss has been highlighted (Page 18):

The first term in Eq. (4), i.e., generalized FAPE loss (gFAPE), quantifies the deviation between the predicted global nucleotide-wise rigid transformation ($T$) and the experimental global nucleotide-wise rigid transformation ($T^{exp}$), which is defined as,

$$\begin{aligned} gFAPE\left( T,T^{exp},\lambda,d^{cut} \right)= \\ \sum_{i} \sum_{j} \lambda_{ij}\min\left( d_{ij}^{cut},\sqrt{\left\| T_{i}^{-1}\left( T_{j}\left( \vec{r} \right) \right)-{T_{i}^{exp}}^{-1}\left( T_{j}^{exp}\left( \vec{r} \right) \right) \right\|^{2}+\epsilon} \right)\#\left( 8 \right) \end{aligned}$$

where $\lambda_{ij}$ represents the inter-nucleotide re-weighting factor and $d_{ij}^{cut}$ denotes the deviation cut-off parameter between nucleotides *i* and *j*.

With these upgrades, DRfold2 substantially outperforms DRfold on our benchmark (S10 Fig).

**Response to Reviewer #3**

We very much appreciate the comments and suggestions from the Reviewer, which help to significantly improve the quality of the manuscript. The major concern from this Reviewer is the interpretability of RCLM and possible data leakage problem. In the updated version, we have performed additional experiments to address the Reviewer’s concerns. Below, we include point-by-point replies to the comments of the Reviewer, with all changes highlighted in yellow.

**1. The Reviewer commented:**

***The manuscript introduces DRfold2, a deep learning framework for ab initio RNA structure prediction. Key innovations include: (1) A novel RNA Composite Language Model (RCLM) trained using composite likelihood maximization to capture co-evolutionary patterns from unannotated sequences. (2) A Denoising RNA Structure Module (DRSM) for end-to-end 3D structure prediction. (3)A CSOR protocol for model selection/optimization. DRfold2 claims superiority over state-of-the-art methods (e.g., trRosettaRNA, RhoFold, AlphaFold3) in global***

***topology (TM-score) and secondary structure (INF metrics) across multiple benchmarks. It also highlights complementarity with AlphaFold3, showing improved accuracy when integrated. However, the intellectual contribution to understanding RNA 3D structure folding is not clear. And the training process may suffer from data leakage. Major and minor comments are listed as below.***

We thank the Reviewer for the summary of the manuscript.

**2. The Reviewer commented:**

***Major Concerns:***

***1. The paper positions DRfold2 as a technical advance but fails to derive new RNA folding principles from the model. For example: Does RCLM reveal specific nucleotide interaction rules (e.g., tertiary motifs, ion-binding preferences)? How do denoising or composite likelihood strategies translate to biophysical knowledge? I recommend including analysis of RCLM attention weights or feature importance to extract interpretable biological rules.***

We very much thank the Reviewer for pointing this out. RCLM indeed provides folding principles by capturing co-evolutionary dependencies that correlate strongly with physical interactions, as demonstrated in Sections ***RCLM more effectively captures co-evolutionary patterns than existing language models*** and ***Positive impact of composite language model on RNA 3D structure prediction***. The co-evolution coupling signals can accurately predict experimentally observed interaction patterns, as shown in **Fig 5**. However, whether RCLM also learns other tertiary motifs or ion-binding preferences remains an open question and warrants further investigation.

According to the Reviewer’s suggestion, we performed an attention-based analysis to investigate how RCLM learns physical interactions. We have added a new section titled ***RCLM learns base pairing interactions in an unsupervised manner***. The first two paragraphs read as follows:

***RCLM learns base pairing interactions in an unsupervised manner***

Although the model is optimized purely for likelihood-based sequence reconstruction, the coevolutionary statistics it captures are validated to correlate strongly with physical contacts (**Fig 5**). To further understand how RCLM learns these physical interactions, we conducted a nucleotide-masking experiment (in addition to the Categorical Jacobian analysis). Specifically, for each annotated base-pairing interaction, we masked one nucleotide and assessed how accurately RCLM could recover the masked residue from the remaining context, and the corresponding attention weight distributions. Representative examples are shown in **S6 Fig** for two targets whose PDB IDs are 8HZD (chain A) and 8Z8U (chain A) respectively. In 8HZD_A, masking positions 2 or 6 causes the attention mechanism to assign higher weights to their long-range canonical and non-canonical partners, indicating that RCLM learns these interactions by attending directly to the physically paired nucleotides. A similar behavior is observed in 8Z8U_A for positions 1 and 20. However, this physically grounded attention does not always generalize, especially for some non-canonical interactions. For instance, when masking position 8 in 8HZD_A or position 13 in 8Z8U_A, the model relies more on local neighboring nucleotides rather than attending to the true long range interaction partner.

Overall, for canonical Watson–Crick pairs, the recovery success rate was 57.1%, indicating that RCLM effectively captures canonical interacting patterns. However, for non-canonical interactions, the recovery rate dropped to 36.6%, suggesting that RCLM learns weaker signals for these interaction types (**S7 Fig**). Both canonical and non-canonical base pairs are recovered at higher accuracies above random expectation (~25%), indicating that RCLM has learned to recognize true physical pairing signals without any structural supervision.

**S6 Fig. Examples of attention-based nucleotide-masking analysis for canonical and non-canonical RNA interactions.** **(A)** Results for **8HZD chain A**. **(B)** Results for **8Z8U chain A**. Nucleotides in red indicate the masked positions. Green nucleotide boxes represent attention magnitudes, where deeper green corresponds to higher attention weights. Blue arcs denote canonical base pairs, red arcs denote non-canonical pairs, and magenta arcs highlight interactions involving the masked nucleotide. The attention weights are extracted from the Attention module within the final RNA Transformer Block.

**S7 Fig. Recovery rates of canonical and non-canonical interactions by RCLM.**

Overall, our analyses show that RCLM learns both canonical and non-canonical interactions without any structural supervision, and these learned interaction patterns provide DRfold2 with richer sequence-derived constraints that guide tertiary structure folding. The attention-based analysis further clarifies how RCLM captures these long-range interactions by attention mechanism.

**3. The Reviewer commented:**

***2. The model training process is likely to suffer from data leakage. Both the training set and the test set are redundant. E.g., 2 CPEB3 ribozymes are in the test set; multiple tRNAs with very similar sequences are found in the training set. In particular, PreQ1 Riboswitch, CoV-SL5, Sars-Cov-2 FSE exist in both training set and test set. Therefore, the model training is not reliable and all these benchmark results cannot be trusted.***

***Technically speaking, the test set includes PDB entries after 2024 (e.g., 9KPO, released March 2025), but RCLM was trained on RNAcentral (release 22, circa 2022-2023). However, no validation that post-2024 structures (or their close homologs) were absent from training. Sequence identity cutoff (80%) is insufficient; structural homology could persist below this threshold.***

We thank the Reviewer for raising this important concern regarding potential data leakage.

First, we agree that sequence identity alone cannot fully guarantee the absence of structural redundancy, as distinct sequences may still adopt highly similar folds. Nevertheless, since DRfold2 is trained to learn a mapping from sequence to structure, ensuring that the training and test sequences are different should, in theory, prevent the model from relying on trivial memorization of previously observed samples; if the model still performs well on sequences not seen during training, this indicates that it has learned generalizable sequence–structure relationships rather than simply reproducing stored examples.

However, it is also important to evaluate whether DRfold2 can generalize to structurally novel folds, as the Reviewer rightly pointed out. To address this, we performed an additional experiment based on structural homology filtering, Accordingly, we have added the results of this analysis as a separate section (Page 6):

***DRfold2 enables robust RNA structure prediction for targets with novel folds***

While DRfold2 shows highly competitive performance against existing top-performing methods such as AlphaFold3, it is also of interest to examine how DRfold2 performs on targets whose structural folds differ from those present in the training set. We utilized US-align [37] to filter out targets that have maximum normalized TM-score > 0.45 against all training samples. This procedure yields a total of 25 novel-fold targets.

On these novel folds, DRfold2 achieves an average TM-score of 0.309, which is lower than its performance on the full benchmark set (0.350), indicating that prediction accuracy decreases for unseen structural topologies. Importantly, DRfold2 is trained to learn a mapping from sequence to structure, and for targets with distinct sequences, the model cannot rely on simple memorization of previously observed structures to produce correct predictions. The generally higher performance of DRfold2 on those known-fold targets over novel targets, actually demonstrates that the DRfold2 model can capture latent structural representations that go beyond sequence similarity.

Notably, DRfold2 consistently outperforms AlphaFold3 on this subset of new-fold RNAs (0.309 vs. 0.281), with a p-value of 5.29E-02 for one-tailed t-test (**S2 Fig**). Furthermore, DRfold2 achieves a significantly lower RMSD (15.888 vs. 18.832) with a p-value of 1.97E-02 (one-tailed t-test), despite the limited number of samples. These results suggest that DRfold2 can better retain nontrivial generalization ability by extracting structural principles from sequence and does not simply memorize previously seen folds or sequences.

**S2 Fig.** **TM-score and RMSD comparisons between DRfold2 and AlphaFold3 on the novel-fold RNA targets.** **(A)** TM-score comparison. **(B)** RMSD comparison.

**4. The Reviewer commented:**

***3. The results provide inadequate statistical support. E.g., need statistical tests and p-values for P4L158, P4L164, P6L248, P7L296, P8L321, P8L329, P8L354, P9L391, P10L423, P10L433, P10L449, P22L825.***

We thank the Reviewer for the careful reading. We have revised all places where the term “significant” was previously used imprecisely. Specifically, we corrected wording in the following locations:

P4L158 now reads:

In **Fig 2C**, we present one example of a CPEB3 HDV-like ribozyme from Chimpanzee (PDB ID: 7QR3), a 69-nucleotide RNA molecule, where DRfold2 shows advantage in modeling tertiary structure.

P4L164 now reads:

RhoFold shows errors in predicting the spatial organization of the junction regions, with TM-scores reducing to 0.323. (“significant” removed)

P7L296 now reads:

Collectively, these results suggest that the comprehensive representations provided by advanced language models, like RCLM, can enhance the ability to learn co-evolutionary patterns and spatial restraints, leading to more accurate 3D RNA structure modeling through the end-to-end network in DRfold2. (“significant” removed)

P8L321 now reads:

In contrast, the 5^th^-ranked model from DRfold2 successfully predicted the junction’s direction, reducing the RMSD from 22.05 Å (first DRfold2 model) to 6.54 Å (right panel of **Fig 3C**). with significant removed

P8L354 now reads:

The results revealed that Vfold, which utilizes a template-based modeling approach integrated with AlphaFold3 [51], achieved the highest performance, with an average TM-score of 0.566 and Z-score of 19.71, outperforming other groups. (“significant” removed)

P10L423 now reads:

While it shares a similar name with the earlier DRfold method [21], DRfold2 introduces key advancements built on a completely different framework, most notably with the incorporation of the Composite Language Model, which greatly enhances RNA sequence and pairwise representations.

P10L449 now reads:

It is worth noting that two sub-modules, i.e., Triangle Attention StartingNode and Triangle Attention EndingNode modules, were omitted in RCLM, as this configuration can reduce the GPU memory consumption. (“significant” removed)

P22L825 now reads:

Note that the mean Deformation Indexes for trRosettaRNA and RhoFold are much higher than the median, due to their poor performance on a few targets where no correctly modeled base pairs are present in the predicted structures.

For other cases where “significant” was corrected placed, we have added statistical justifications.

P6L248 now reads:

RCLM, RCLM-M, and RiNALMo exhibit comparable performance, while RCLM once again achieves highest precision and shows a statistically significant advantage over the second-highest model, RiNALMo, in Top-*L*/2 precision, with p-value of 8.57E-03 in one-tailed t-test.

P9L391 was retained because statistical significance was already established in the main text (Section ***RCLM more effectively captures co-evolutionary patterns than existing language models***) (Page 9):

RCLM also produces significantly higher contact prediction than the second best LM model, RiNALMo, with p-values of 4.82E-09, 5.00E-09, and 1.04E-09 for thresholds of 12, 16 and 20 Å, respectively, in one-tailed t-test.

P10L433 now reads:

Equipped with those advancements, DRfold2 significantly outperforms DRfold (**S10 Fig)** in TM-score (0.350 versus 0.300) with p-value of 8.03E-04 in one-tailed t-test, providing 2.5 times as many (from 4 to 10) foldable RNAs (TM-score > 0.45) on the test set used in this study.

**5. The Reviewer commented:**

***4. There should be clearer comparison with AlphaFold3 in Figures 2 and 3. There is no analysis of specific RNA categories (e.g., pseudoknots, riboswitches) where DRfold2 excels/fails vs. AlphaFold3. And the complementarity claims (Fig. 6) lack mechanistic explanation.***

We are grateful for the constructive comments by the Reviewer.

(1) We have added AlphaFold3 to both **Fig 2** and **3** (now **Figs 2** and **4** in revised manuscript). Specifically, we have moved the comparison between DRfold2 and AlphaFold3 into the main comparison section ***DRfold2 outperforms existing methods in global RNA tertiary structure prediction***. the following contents are added to the first paragraph of the section (Page 3):

We also compare DRfold2 with the latest iteration of AlphaFold, AlphaFold3 [31], for RNA 3D structure prediction. For doing this, we submitted the RNA sequences in our test set to the AlphaFold Server and obtained the predicted structures of AlphaFold3 with default seed configurations.

We have also revised the second paragraph of this section (Page 4) to incorporate the performance of AlphaFold3 together with other control methods. The updated text now reads:

Within the test dataset, there are 9, 21, 37, and 41 targets with sequence identities below 50%, 60%, 70%, and 80%, respectively, relative to the training dataset. In general, prediction accuracy of DRfold2 increases for the targets with higher sequence identity to the training set. Notably, this trend holds for all control methods even though no filters was applied to their training dataset, probably because these methods were trained on RNAs released before 2024 which have a similar homologous distance to the new test dataset used in this study. It is observed that DRfold2 consistently achieves the highest average TM-score among all methods across all sequence identity thresholds. For example, at the 80% cutoff, DRfold2 attains an average TM-score of 0.350 which is 19.9% higher than DeepFoldRNA (TM-score=0.292). The difference is statistically significant, with a p-value of 6.37E-04 in the one-tailed Student’s t-test, despite the low degree of freedom, i.e., the limited sample size of 41. Even with the most stringent test subset containing 9 test targets (50% sequence identity cutoff), DRfold2 achieves an average TM-score of 0.275, which is 20.0% higher than RoseTTAFoldNA (TM-score=0.229), with a p-value of 1.43E-02 (one-tailed t-test). Meanwhile, DRfold2 exhibits a lower average RMSD than all other control methods across different sequence identity thresholds. DRfold2 also achieves a slightly higher TM-score (0.350) and lower RMSD (14.40 Å) than AlphaFold3 (0.347 and 16.00 Å) (**S1 Table**); however, the difference between the two is not statistically significant, with p-values of 8.91E-01 and 1.37E-01 for TM-score and RMSD respectively, in two-tailed Student’s t-test.

We have updated **Fig 2** and **Fig 3** to include both AlphaFold3 and the hybrid DRfold2+AlphaFold3 potential (denoted DRAF). The corresponding descriptions are 4^th^ and 5-7^th^ paragraphs, respectively, in Section ***DRfold2 outperforms existing methods in global RNA tertiary structure prediction***:

4^th^ paragraph for Fig 2:

In **Fig 2C**, we present one example of a CPEB3 HDV-like ribozyme from Chimpanzee (PDB ID: 7QR3), a 69-nucleotide RNA molecule, where DRfold2 shows advantage in modeling tertiary structure. Among the four better-performing methods in our benchmark, DRfold2 accurately captures the topology of the ribozyme with a TM-score and RMSD of 0.588 and 2.72 Å, respectively. In contrast, DeepFoldRNA and AlphaFold3, while capturing the general helical arrangement, show notable deviations in the orientation of the hairpin loop. As a result, the RMSD becomes 5.68 Å and 7.98 Å respectively, which is at least twice the deviation of DRfold2. RhoFold shows errors in predicting the spatial organization of the junction regions, with TM-scores reducing to 0.323. Note that the maximum sequence identity between this target and the training RNAs is only 60.9%, demonstrating that the DRfold2 pipeline can provide reasonable structure predictions even for novel sequences that are not homologous to the training data.

5-7^th^ paragraphs for Fig 3:

**Figs 3A&B** provide head-to-head TM-score and RMSD comparisons by the DRfold2 and AlphaFold3. Although DRfold2 outperforms AlphaFold3 in several low- to medium-quality models, AlphaFold3 excels in two targets with TM-score above 0.6. A detailed comparison between DRfold2 and AlphaFold3 across different target annotations is shown in **S2 Table**, where pseudoknots were annotated using RNApdbee [34] and all other categories follow the official NAKB annotations [35]. DRfold2 achieves a higher average TM-score on targets containing pseudoknots, suggesting that it produces more accurate global folds in these cases. However, in terms of pseudoknot recovery rate, DRfold2 is notably lower than AlphaFold3, i.e., 56.3% versus 79.1%, indicating that AlphaFold3 captures atomic-level base-pairing conformations more accurately. For reference, other control methods have substantially lower pseudoknot recovery rates, i.e., 19.0% and 2.0% for RoseTTAFoldNA and RhoFold, respectively. For the remaining NAKB-defined categories, DRfold2 performs better on Aptamer, Catalytic, and G-quadruplex RNAs, whereas AlphaFold3 shows advantages on Virus and Riboswitch targets.

While most of our above discussions focus on the top-rank models by DRfold2, we have identified several targets for which DRfold2 generated correct structures, but the post-process step failed to select them as the first-rank models. **Fig 3C** illustrates one such examples from the coxsackievirus B3 cloverleaf RNA (PDB ID: 8DP3), which contains 90 residues forming a 4-way junction fold [36]. By checking the first-rank models, both DRfold2 and AlphaFold3 failed to correctly predict the direction of the 4-way junction, with RMSDs to the native around 20 Å. To further explore AlphaFold3's capabilities, we submitted the same sequence to AlphaFold Server 20 times with different seeds, resulting in 100 predicted conformations. The middle panel of **Fig 3C** shows the best conformation with the highest TM-score, which still exhibits a poor RMSD of 21.83 Å due to incorrect chain winding topology across the 4-way junction. In contrast, the 5^th^-ranked model from DRfold2 successfully predicted the junction’s direction, reducing the RMSD from 22.05 Å (first DRfold2 model) to 6.54 Å (right panel of **Fig 3C**). While this example highlights DRfold2’s capability to generate diverse and alternative conformations, it also underscores the need to improve the post-processing CSOR protocol for more effectively selecting and ranking the correct models from the DRfold2 decoy pool, e.g., through the combination of third-party algorithms.

Although DRfold2 and AlphaFold3 exhibit similar overall performance, the results in **Figs 3A-B** highlight the strong complementarity between the two programs, particularly in cases where predictions deviate from the diagonal line. This complementary behavior, along with the identified need to improve DRfold2’s model selection strategy, motivated us to explore whether combining the two approaches could lead to enhanced structure predictions. The optimization module in DRfold2 readily facilitates this integration by incorporating AlphaFold3 conformations as an additional potential term. Guided with this combined potential function, the optimized structures achieve higher quality models than those generated by either method alone, as shown in **Fig 2** labelled as DRAF, and in the detailed head-to-head comparison in **Figs 3D&E**. The hybrid approach achieves an average TM-score of 0.368 and RMSD of 14.02 Å, both being significantly better than that of AlphaFold3 (0.347 and 16.00 Å, with p-value = 4.86E-02 for TM-score and 3.16E-03 for RMSD, one-tailed t-test). These results not only validate our hypothesis regarding the complementarity between DRfold2 and AlphaFold3 but also demonstrate the flexibility and extensibility of the CSOR module, enabling the seamless integration of external model features to further improve structure prediction in DRfold2.

.

**Fig 2. RNA structure prediction results on the test dataset by different methods across different sequence identity thresholds. (A)** Box plots of TM-score and RMSD for eight RNA structure prediction methods evaluated at different sequence identity cutoffs (50-80%) between training and test datasets, with green point and white horizontal line representing the mean and median values respectively. **(B)** Cumulative distribution plots of TM-score and RMSD for the predicted models. Upper panels display the fraction of models above different TM-score thresholds, while lower panels show the fraction of models below different RMSD values. **(C)** A representative modeling example from Chimpanzee CPEB3 HDV-like ribozyme (PDB ID: 7QR3), with models predicted by four better-performing methods (blue cartoons) overlaid on experimental structure (gray cartoons).

**Fig 3. Comparative analysis of DRfold2 and AlphaFold3 for RNA structure predictions. (A-B)** Head-to-head TM-score and RMSD comparisons between DRfold2 and AlphaFold3. **(C)** Structural visualization of the example from coxsackievirus B3 cloverleaf RNA (PDBID: 8DP3), showing experimental structure, AlphaFold3's best prediction from 100 models, and 5^th^ model of DRfold2, respectively. Structures are rainbow-colored from 5' (blue) to 3' (red) end. **(D-E)** TM-score and RMSD comparisons between AlphaFold3 and DRfold2 with DRfold2+AlphaFold3 potential. **(F)** Performance comparisons between DRfold2 and Alphafold3 grouped by MSA depth retrieved by AlphaFold3. Green points indicate means and white horizontal lines show medians.

In addition, the comparisons of RNA secondary structures have also been updated in Section ***DRfold2 networks enhance the accuracy of RNA secondary structure prediction*** (Page 8):

Many RNA functions are closely associated with their secondary structure arrangements, which are primarily stabilized by hydrogen bonding and base-pair stacking interactions [42]. **Figs 4A-D** analyzes the Interaction Network Fidelity (INF) indices, which are defined as the geometric mean of precision and recall of secondary structure predictions, i.e., $INF=\sqrt{precision\times recall}$. First, for Watson-Crick base pairs (INF_wc) and stacking interactions (INF_stack), while most methods (except for trRosettaRNA) demonstrate reasonable performance, DRfold2 achieved the INF values of 0.831 and 0.754, slightly lower than those of AlphaFold3 (0.849 and 0.762), but higher than all other control methods. For non-canonical interactions (INF_nwc), however, all methods, including DRfold2 and AlphaFold3, exhibit much lower INF values. Although DRfold2 and AlphaFold3 still achieves the top-2 INF_nwc, their low value (0.184 and 0.202, respectively) underscores that accurately modeling non-canonical base-pair interactions remains an urgent challenge in RNA structure prediction. **Fig 4D** presents a combined secondary structure accuracy across all three indices. DRfold2 achieves INF_all = 0.750, which is statistically comparable to that of AlphaFold3 (p-value = 2.92E-01, two-tailed t-test), and 7.4% higher (p-value = 5.97E-04, one-tailed t-test) than the third-best method, DeepFoldRNA (INF_all = 0.698).

While the INF indices assesses the conformational accuracy at the nucleotide pair level, the Deformation Index (DI), defined as $RMSD/INF\_all$, has been introduced to provide a more balanced evaluation of 3D topological and 2D base-pairing patterns [43]. As shown in **Fig 4E**, DRfold2 achieves the lowest average DI of 20.21, which is 9.6% lower than the second-best method, AlphaFold3 (DI=22.15), The corresponding p-value of 1.43E-01 in two-tailed t-test indicates that this improvement is not statistically significant. However, the method that combines DRfold2 and AlphaFold3 potentials (DRAF) achieves the lowest DI of 20.15, which is significantly lower than that of AlphaFold3 (p-value = 2.14E-02, one-tailed t-test). **Fig 4F** further presents the accumulative fraction distribution at different DI cut-offs, where DRfold2 outperforms all third-party control methods, achieving the highest normalized area under the curve (AUC=0.520), which is 5.3% higher than the best control method, AlphaFold3 (AUC=0.494). These results suggest that DRfold2 excels not only in predicting global structural topology but also in capturing detailed hydrogen-bonding and base-pair stacking patterns within RNA structures.

(2) Following Reviewer’s suggestion, we have compared DRfold2 and AlphaFold3 across different functional and structural annotations. We have added the following paragraph in Section ***DRfold2 outperforms existing methods in global RNA tertiary structure prediction*** (Page 4-5):

**Figs 3A&B** provide head-to-head TM-score and RMSD comparisons by the DRfold2 and AlphaFold3. Although DRfold2 outperforms AlphaFold3 in several low- to medium-quality models, AlphaFold3 excels in two targets with TM-score above 0.6. A detailed comparison between DRfold2 and AlphaFold3 across different target annotations is shown in **S2 Table**, where pseudoknots were annotated using RNApdbee [34] and all other categories follow the official NAKB annotations [35]. DRfold2 achieves a higher average TM-score on targets containing pseudoknots, suggesting that it produces more accurate global folds in these cases. However, in terms of pseudoknot recovery rate, DRfold2 is notably lower than AlphaFold3, i.e., 56.3% versus 79.1%, indicating that AlphaFold3 captures atomic-level base-pairing conformations more accurately. For reference, other control methods have substantially lower pseudoknot recovery rates, i.e., 19.0% and 2.0% for RoseTTAFoldNA and RhoFold respectively. For the remaining NAKB-defined categories, DRfold2 performs better on Aptamer, Catalytic, and G-quadruplex RNAs, whereas AlphaFold3 shows advantages on Virus and Riboswitch targets.

**S2 Table. Detailed comparison of AlphaFold3 and DRfold2 for different categories grouped by function and structure annotations.** Bold fonts highlight better performance in each subcategory.

| Annotations | AlphaFold3 | DRfold2 |
| --- | --- | --- |
| Pseudoknot (n = 20) | 0.385 | **0.393** |
| Aptamer (n = 5) | 0.238 | **0.302** |
| Virus (n = 6) | **0.448** | 0.436 |
| Riboswitch (n = 8) | **0.382** | 0.354 |
| Catalytic (n = 8) | 0.342 | **0.386** |
| G-quadruplex (n = 2) | 0.228 | **0.284** |

(3) We explain the complementarity between DRfold2 and AlphaFold3 through their distinct dependencies on evolutionary information as reflected by MSA depth. Accordingly, we have added the following analysis (Page 6-7):

***DRfold2 enables robust RNA structure prediction with few homologues***

Next we examine how the performance of DRfold2 varies across different levels of evolutionary information, as reflected by the MSA depth retrieved by AlphaFold3. In **Fig 3F**, we present this comparison together with AlphaFold3. For reference, we also include the performance of DRAF as well as that of AlphaFold3 without MSA input. In general, both DRfold2 and AlphaFold3 achieve better performance with the increase of numbers of homologous sequences, likely due to richer co-evolutionary constraints. When no MSA is available, AlphaFold3 achieves an average TM-score of only 0.333, which is moderately lower than its full-version performance (0.347).

Although DRfold2 and AlphaFold3 show comparable overall accuracy, their behavior differs across MSA-depth groups. For targets without any detectable homologs (MSA depth = 0), DRfold2 achieves slightly higher accuracy (TM-score = 0.300) compared to AlphaFold3 (TM-score = 0.287). A similar trend is observed for low-homology targets (MSA depth 1–10), where DRfold2 outperforms AlphaFold3 with TM-scores of 0.442 versus 0.407. For targets with richer evolutionary information (for example, MSA depth 11–20), however, AlphaFold3 performs better (TM-score = 0.357 versus 0.311 for DRfold2). Nonetheless, the hybrid folding pipeline, DRAF, which integrates DRfold2 predictions with the AlphaFold3 potential, achieves the highest accuracy (TM-score = 0.368) in this group, demonstrating the complementarity of the two approaches.

Taking together, these results further highlight the complementary strengths of DRfold2 and AlphaFold3. DRfold2 is trained primarily on single-sequence features enriched with co-evolutionary signals derived from RCLM. The hidden universal covariance learned by RCLM becomes more valuable, for example, when explicit homologues are insufficient or not detected. Consequently, DRfold2 can provide more reliable predictions for RNAs with few or even no homologues.

**Fig 3. (F)** Performance comparisons between DRfold2 and Alphafold3 grouped by MSA depth retrieved by AlphaFold3. Green points indicate means and white horizontal lines show medians.

**6. The Reviewer commented:**

***5. The contribution of RCLM to non-canonical interactions is unclear. RCLM improves Watson-Crick pairs (INF_wc=0.838) but performs poorly on non-canonical pairs (INF_nwc=0.121, Fig. 3). The manuscript should quantify RCLM's role in non-canonical accuracy. Why it can improve? The authors should also discuss if composite likelihood specifically aids non-Watson-Crick modeling.***

We thank the Reviewer for pointing out the interesting questions.

(1) Regarding the RCLM’s contribution to modeling canonical and non-canonical pairs, our analyses show that RCLM can learn both canonical and non-canonical base-pairing relationships without any structural supervision, and these signals are among the key features used by DRfold2. The difference in recovery success rate in the nucleotide-masking experiment may partly explain why DRfold2 achieves higher performance on canonical base-pair modeling compared to non-canonical ones. Accordingly, we have added the following descriptions in Section ***RCLM learns base pairing interactions in an unsupervised manner*** (Page 11):

Overall, for canonical Watson–Crick pairs, the recovery success rate was 57.1%, indicating that RCLM effectively captures canonical interacting patterns. However, for non-canonical interactions, the recovery rate dropped to 36.6%, suggesting that RCLM learns weaker signals for these interaction types (**S7 Fig**). Both canonical and non-canonical base pairs are recovered at higher accuracies above random expectation (~25%), indicating that RCLM has learned to recognize true physical pairing signals without any structural supervision. Such difference in accuracy may partly account for the higher performance of DRfold2 on canonical, compared to non-canonical, base-pair modeling (**Fig 4B**).

**S7 Fig. Recovery rates of canonical and non-canonical interactions by RCLM.**

(2) Regarding whether composite likelihood specifically benefits non-canonical interactions, we performed a two-nucleotide masking experiment on annotated non-canonical pairs. Overall, the additional pairwise head achieved a slightly higher recovery rate than the sequence head. However, the difference is not statistically significant, suggesting that the pairwise terms in composite likelihood do not provide a clear advantage for non-canonical interaction modeling. Detailed descriptions and explanations have been added to Section ***RCLM learns base pairing interactions in an unsupervised manner*** (Page 11):

We further performed a two-nucleotide masking experiment on annotated non-canonical pairs. In this setting, both nucleotides in a non-canonical interaction were masked, and we evaluated whether RCLM could correctly recover both nucleotide identities using the sequence-level prediction head and the pairwise prediction head (supervised by the pairwise likelihood term). Overall, the pairwise head achieved a slightly higher recovery rate (18.0%) compared with the sequence head (17.8%). The pairwise head outperformed the sequence head in 9 targets, while the sequence head outperformed the pairwise head in 5 targets. However, the difference is not statistically significant (p-value = 7.81E-01, two-tailed t-test), indicating that the composite likelihood does not provide a significant advantage for non-canonical interactions. This observation, however, aligns with our expectation since all nucleotide pairs are treated equally without bias terms for non-canonical pairs during the RCLM training.

In summary, these results confirm that, while RCLM provides strong support for canonical base-pair learning, its ability to model non-canonical interactions remains limited. Nevertheless, they also suggest potential directions for improvement, such as explicitly modeling non-canonical interactions in composite likelihood.

**7. The Reviewer commented:**

***Minor Concerns:***

***The authors can still improve technical clarity.***

***P21: The noise-injection protocol is vague. Specify how w=0.1 and ϵ∼N(0,0.1) were chosen.***

We thank the Reviewer for the constructive comments. We explain the parameter configuration in Section ***End-to-end RNA structure prediction with the Denoising Structure Module*** (Page 17):

The parameters $w$ and $\varepsilon$ were chosen conservatively to introduce minor perturbations to the input without destroying the structural signal.

**8. The Reviewer commented:**

***P22: CSOR protocol: Clarify why base-pair count filtering (>150 nt) stabilizes optimization.***

We thank the Reviewer for the comment. Filtering by base-pair count helps select more structurally regular and more accurate conformations, which improves the stability of the subsequent optimization step, especially for long RNAs. We have added the detailed explanation in Section ***Structure optimization through CSOR protocol*** (Page 19):

We observed that the top 10% of conformations ranked by base-pair count generally have higher average TM-scores (**S12 Fig**). This improvement is more pronounced for targets longer than 150 nt, with a ΔTM-score of 0.026, compared to 0.012 for shorter targets. We hypothesize that filtering by base-pair count preferentially selects conformations that better capture regularized structural motifs, thereby stabilizing the optimization.

**S12 Fig. TM-score improvements by filtering based on base pair counts across targets for different lengths.**

**9. The Reviewer commented:**

***P15: Categorical Jacobian: Briefly define how it extracts contacts.***

We thank the Reviewer for the comment. We have added a separate Section to explain how to extract contacts using Categorical Jacobian (Page 16):

***Extracts contact score with Categorical Jacobian***

Once the RCLM is trained, we can obtain a function $f(X)$ that maps an RNA sequence of length *L* into a position-wise distribution matrix with a shape of $L\times4$. Based on that, we construct a coupling tensor *J* with a shape of $L\times4\times L\times4$, defined as:

$$\begin{aligned} J_{i,a,j,b}=\left[ f\left( X\left( x_{i}\to N_{a} \right) \right) \right]_{j,b}-\left[ f\left( X \right) \right]_{j,b}\#\left( 3 \right) \end{aligned}$$

Here, $x_{i}\to N_{a}$ means performing a mutation at *i*-th position into *a*-th nucleotide type $N_{a}$.

We then transform the coupling matrix *J* into an intermediate $L\times L$ contact matrix *M* by summing the squared values over the nucleotide dimension:

$$\begin{aligned} M_{i,j}=\left( \sum_{a=1}^{4} \sum_{b=1}^{4} \left( J_{i,a,j,b} \right)^{2} \right)^{\frac{1}{2}}\#\left( 4 \right) \end{aligned}$$

Finally, the Average Product Correction (APC) is then applied to remove the background noise and to obtain the final contact map.

$$\begin{aligned} C_{i,j}=M_{i,j}-\frac{\sum_{i^{'}}^{L} M_{i^{'},j}\sum_{j^{'}}^{L} M_{i,j^{'}}}{\sum_{i^{'}}^{L} \sum_{j^{'}}^{L} M_{i^{'},j^{'}}}\#\left( 5 \right) \end{aligned}$$

**10. The Reviewer commented:**

***Typos and Inconsistencies:***

***Abstract: "denoise-based" should be "denoising-based".***

***P4L143: "despite the low degree of freedom" → specify degrees of freedom.***

***P18L746: "Rinalmo" → "RiNALMo" (throughout).***

***P8L324: "SCOR" → "CSOR" (inconsistency with Fig. 1E).***

***Ref 22 (Li et al., 2022): avoid <em> in the text.***

We thank the Reviewer for the careful proofreading. The phrase “low degree of freedom” refers to the limited sample size used in the statistical test, and we have clarified this in the revised manuscript. All other noted typos and inconsistencies have also been fixed accordingly.
