# Supplementary material for: DRfold2 is a deep learning-based tool that enables efficient and accurate RNA structure prediction
Source: PLoS Biol. 2026 Feb 17;24(2):e3003659. doi: 10.1371/journal.pbio.3003659 (PMC12931758; doi:10.1371/journal.pbio.3003659)
Supplement: Attachment — Submitted filename: Response to Reviewers R2.docx [file pbio.3003659.s022.docx]

**Response to Reviewer #1**

1. **The Reviewer commented:**

***I have not further comments.***

We are glad that the Reviewer is satisfied with our Revision.

**Response to Reviewer #2**

2. **The Reviewer commented:**

***The authors have resolved my previous concerns.***

We are glad that the Reviewer is satisfied with our Revision.

**Response to Reviewer #3**

We very much appreciate the insightful comments and suggestions from the Reviewer, which have significantly improved the clarity and presentation of the manuscript. In the revised version, we have added clarifications to explain several unclear concepts and rationales pointed out by the Reviewer. Below, we include point-by-point replies to the comments of the Reviewer, with all changes highlighted in yellow.

**1. The Reviewer commented:**

***I appreciate the authors' careful and detailed responses to the previous round of comments, as well as the additional analyses that have been incorporated into the revised manuscript. The revision has substantially improved the clarity of the work. Some conceptual and methodological points would benefit from further clarification to ensure that the conclusions drawn are fully supported.***

We thank the Reviewer for accepting our previous responses.

**2. The Reviewer commented:**

***1. Line 246: Demonstrating performance on targets with normalized TM-score ≤ 0.45 relative to the training set strengthens the argument that DRfold2 does not rely solely on trivial memorization. Nevertheless, regarding the inclusion of post-2024 PDB structures, a brief clarification of whether homologous sequences or highly similar families might already exist in RNAcentral (release 22) would improve transparency, even if full exclusion cannot be guaranteed.***

We thank the Reviewer for this thoughtful suggestion. We would like to clarify that the RNA composite language model is trained in a fully self-supervised manner using sequence information only, without incorporating any experimentally resolved structural information. As the Reviewer correctly points out, given the scale of RNAcentral (release 22), homologous sequences or closely related RNA families to some post-2024 PDB targets may already exist in the sequence training corpus, and full exclusion cannot be guaranteed. We have added the following explicit clarification to the manuscript to improve transparency (Page 6):

While DRfold2 shows highly competitive performance against existing top-performing methods such as AlphaFold3, it is also of interest to examine how DRfold2 performs on targets whose structural folds differ from those present in the training set. We utilized US-align [37] to filter out targets that have maximum normalized TM-score > 0.45 against all training samples. This procedure yields a total of 25 novel-fold targets. We note that, while structurally homologous folds are excluded by this procedure, homologous sequences or closely related RNA families may still exist in the large-scale sequence corpus (RNAcentral, release 22) used for language model pretraining, which is unavoidable in self-supervised sequence modeling. However, no experimentally resolved structural information from these sequences is used during training, and the novel-fold analysis therefore does not imply reliance on trivial structural memorization.

**3. The Reviewer commented:**

***2. Line 284: For the hybrid DRAF approach, a brief clarification of whether AlphaFold3 is used purely as an external structural prior or implicitly transferring training knowledge would help avoid confusion about fairness and information flow between models.***

We thank the Reviewer for this helpful suggestion. We have added a clarification in Section ***DRfold2 outperforms existing methods in global RNA tertiary structure prediction,*** at the point where DRAF is firstly mentioned (Page 5), as follows:

Although DRfold2 and AlphaFold3 exhibit similar overall performance, the results in **Figs 3A-B** highlight the strong complementarity between the two programs, particularly in cases where predictions deviate from the diagonal line. This complementary behavior, along with the identified need to improve DRfold2’s model selection strategy, motivated us to explore whether combining the two approaches could lead to enhanced structure predictions. The optimization module in DRfold2 readily facilitates this integration by incorporating AlphaFold3 conformations as an additional potential term. Here, AlphaFold3 is used purely as an external source of candidate conformations for folding potential construction, without transferring model parameters or learned representations. No joint training, fine-tuning, or parameter sharing is performed between the two models. Guided with this combined potential function, the optimized structures achieve higher quality models than those generated by either method alone, as shown in **Fig 2** labelled as DRAF, and in the detailed head-to-head comparison in **Figs 3D&E**.
